# Mechanisms of PTPσ-Mediated Presynaptic Differentiation

**DOI:** 10.3389/fnsyn.2019.00017

**Published:** 2019-05-22

**Authors:** Claire Bomkamp, Nirmala Padmanabhan, Benyamin Karimi, Yuan Ge, Jesse T. Chao, Christopher J. R. Loewen, Tabrez J. Siddiqui, Ann Marie Craig

**Affiliations:** ^1^Djavad Mowafaghian Centre for Brain Health, Department of Psychiatry, University of British Columbia, Vancouver, BC, Canada; ^2^Health Sciences Centre, Kleysen Institute for Advanced Medicine, University of Manitoba, Winnipeg, MB, Canada; ^3^Department of Physiology and Pathophysiology, Rady Faculty of Health Sciences, University of Manitoba, Winnipeg, MB, Canada; ^4^The Children’s Hospital Research Institute of Manitoba (CHRIM), Winnipeg, MB, Canada; ^5^Department of Cellular and Physiological Sciences, Faculty of Medicine, Life Sciences Institute, University of British Columbia, Vancouver, BC, Canada

**Keywords:** synapse, synaptogenesis, LAR-RPTP, phosphatase, adhesion proteins, liprin, scaffolding proteins

## Abstract

Formation of synapses between neurons depends in part on binding between axonal and dendritic cell surface synaptic organizing proteins, which recruit components of the developing presynaptic and postsynaptic specializations. One of these presynaptic organizing molecules is protein tyrosine phosphatase σ (PTPσ). Although the protein domains involved in adhesion between PTPσ and its postsynaptic binding partners are known, the mechanisms by which it signals into the presynaptic neuron to recruit synaptic vesicles and other necessary components for regulated transmitter release are not well understood. One attractive candidate to mediate this function is liprin-α, a scaffolding protein with well-established roles at the synapse. We systematically mutated residues of the PTPσ intracellular region (ICR) and used the yeast dihydrofolate reductase (DHFR) protein complementation assay to screen for disrupted interactions between these mutant forms of PTPσ and its various binding partners. Using a molecular replacement strategy, we show that disrupting the interaction between PTPσ and liprin-α, but not between PTPσ and itself or another binding partner, caskin, abolishes presynaptic differentiation. Furthermore, phosphatase activity of PTPσ and binding to extracellular heparan sulfate (HS) proteoglycans are dispensable for presynaptic induction. Previous reports have suggested that binding between PTPσ and liprin-α is mediated by the PTPσ membrane-distal phosphatase-like domain. However, we provide evidence here that both of the PTPσ phosphatase-like domains mediate binding to liprin-α and are required for PTPσ-mediated presynaptic differentiation. These findings further our understanding of the mechanistic basis by which PTPσ acts as a presynaptic organizer.

## Introduction

A key early step in the formation of a new synapse involves binding between synaptic organizing proteins expressed on the axon of one neuron and the dendrite of another, which triggers clustering of intracellular synaptic proteins in both neurons. The presentation of a single synaptic organizing protein expressed on the surface of a non-neuronal cell is sufficient to induce local clustering of presynaptic or postsynaptic machinery. This is exemplified by the first discovered and best known pair of synaptic organizing proteins, the postsynaptic neuroligins (Scheiffele et al., 2000) and the presynaptic neurexins (Graf et al., 2004). In addition to the neuroligins and neurexins, a variety of other organizing proteins with similar synaptogenic activities have been described (Südhof, 2018). These include the presynaptically-expressed LAR, protein tyrosine phosphatase σ (PTPσ), and PTPδ, which together make up the LAR-RPTP family and interact with postsynaptic NGL-3 (Woo et al., 2009), TrkC (Takahashi et al., 2011), Slitrk1-6 (Takahashi et al., 2012; Um et al., 2014), Il1RAPL1 (Yoshida et al., 2011), IL1RAcP (Yoshida et al., 2012), SALM3, and SALM5 (Mah et al., 2010; Li et al., 2015).

The mechanism by which synaptic organizing proteins signal the formation of a nascent synapse must involve more than simply adhesion. In the case of neurexin, recruitment of intracellular proteins can occur at least under some circumstances without the presence of the neurexin intracellular region (ICR; Gokce and Südhof, 2013), presumably through an unidentified co-receptor. The same does not appear to be true for the LAR-RPTPs, since a version of PTPσ lacking its ICR acted as a dominant-negative suppressor of synaptogenesis (Takahashi et al., 2011). However, the mechanism by which these proteins exert their effects, including which intracellular interacting proteins and/or co-receptors are involved, is poorly understood.

LAR-RPTPs are comprised of extracellular Ig and FNIII domains which mediate binding to postsynaptic NGL-3, TrkC, Slitrks, IL1RAPL1, IL1RAcP, and SALM ligands (Takahashi and Craig, 2013). The LAR-RPTP Ig1 domain also binds chondroitin sulfate and heparan sulfate (HS), interactions that regulate axon growth (Aricescu et al., 2002; Shen et al., 2009). In the context of synaptogenesis, HS competes with TrkC for PTPσ binding (Coles et al., 2014) but HS may mediate the formation of additional synaptogenic complexes as suggested for a PTPσ-glypican-4-LRRTM4 complex (Ko et al., 2015). The other major family of presynaptic organizers, neurexins, are HSPGs (Zhang et al., 2018). It is not yet clear whether HS-mediated LAR-RPTP interaction with neurexins or other axonal co-receptors may contribute to synaptogenic function.

Following the single transmembrane domain, the LAR-RPTPs have a small wedge domain followed by two phosphatase-like domains, termed D1 and D2, of which only D1 is catalytically active (Streuli et al., 1990; Takahashi and Craig, 2013). There are several known enzymatic substrates of the LAR-RPTPs including p250GAP (Chagnon et al., 2010), β-catenin (Müller et al., 1999; Dunah et al., 2005), and N-cadherin (Siu et al., 2007), which could potentially mediate their synaptogenic effects. The D2 domain binds to the scaffolding proteins of the liprin-α family (Serra-Pagès et al., 1995), the GEF/kinase trio (Debant et al., 1996), and to the CASK-interacting proteins caskin1 and caskin2 (Weng et al., 2011).

Whereas there is little evidence for roles of trio (Astigarraga et al., 2010) or caskin (Weng et al., 2011) in synapse development, considerable genetic and functional evidence from multiple systems implicate liprin-α in presynaptic differentiation. The liprin-α sterile alpha motif (SAM) domains bind CASK (Olsen et al., 2005; Wei et al., 2011), liprin-β (Serra-Pagès et al., 1998), mSyd-1a (Wentzel et al., 2013), and PTPσ (Serra-Pagès et al., 1995). The liprin-α coiled coil domains bind RIM (Schoch et al., 2002) and ELKS (Ko et al., 2003) as well as liprin-α itself (Serra-Pagès et al., 1998). Thus, liprin-α may function as a “hub” for recruitment of other presynaptic molecules. The *C. elegans* homolog of liprin-α is required for normal synapse morphogenesis and synaptic transmission, and its loss results in the mislocalization of multiple presynaptic components (Zhen and Jin, 1999). Additionally, the homologs of the LAR-RPTPs and liprin-α interact genetically in the context of synapse formation in both *C. elegans* (Ackley et al., 2005) and *Drosophila* (Kaufmann et al., 2002). The role of the mammalian liprin-α family, which contains four members (termed liprin-α1–liprin-α4) encoded by four separate genes, is less well characterized. Liprin-α2 and liprin-α3 are the most abundant liprin-α isoforms in the brain (Zürner and Schoch, 2009), show different but overlapping expression patterns, and colocalize with synaptic markers (Spangler et al., 2011; Zürner et al., 2011). Knockdown of liprin-α2 leads to defects in presynaptic release as well as reduced localization of several presynaptic components including CASK and RIM (Spangler et al., 2013). Hippocampal neurons from mice with a knockout of liprin-α3 show defects in synaptic vesicle docking, tethering, and exocytosis (Wong et al., 2018). Together, these observations point to liprin-α as an attractive candidate for mediating presynaptic differentiation in response to binding between LAR-RPTPs and their postsynaptic partners.

Here, we used a molecular replacement strategy in which one or more intermolecular interactions were disrupted by domain deletion or point mutagenesis in order to provide insight into the mechanism by which PTPσ signals the formation of a nascent synapse. We find that the ability of PTPσ to mediate the induction of new presynaptic sites through its canonical trans-synaptic partners does not depend on its ability to dephosphorylate targets or to bind HSPGs, but it does require the binding site for liprin-α. Our results also suggest that, contrary to previous reports, binding between PTPσ and liprin-α involves both the D1 and D2 domains of PTPσ.

## Materials and Methods

### DNA Constructs and Viral Vectors

The pFB-shPTP vector used to package AAV6-shPTP was generated based on L315-shCtrlx4 (Gokce and Südhof, 2013), pFB-AAV-GFP-4xshRNA (Zhang et al., 2018), and shRNA sequences against PTPσ (5′-GGCATCATGGGTAGTGATT-3′), PTPδ [5′-GTGCCGGCTAGAAACTTGT-3′ (Dunah et al., 2005)], and LAR [5′-GGCCTACATAGCTACACAG-3′ (Mander et al., 2005)]. This plasmid was packaged into AAV6 by Virovek. AAV6-GFP-4xshRNA called here shCtrl was described previously (Zhang et al., 2018).

The following constructs were described previously: HA-CD4, pLL-CFP (shCtrl-resistant; Zhang et al., 2018), HA-TrkC and TrkC-CFP (both the non-catalytic form; Takahashi et al., 2011). HA-NGL-3 was created based on NGL-3-CFP (Siddiqui et al., 2013) by inserting an HA tag (YPYDVPDYA) following the signal peptide and removing the C-terminal CFP, and V5-CD4 was created from YFP-CD4 (Takahashi et al., 2011) by replacing the YFP tag with V5 (GKPIPNPLLGLDST) following the signal peptide.

pLL3.7-hSyn-V5-PTPσ wild-type (WT), ICR deletion (ΔICR, missing amino acids 974–1530, KLSQ…HYAT), C1142S, 4K4A, D1 deletion (ΔD1, missing amino acids 993–1232, SNLE…EAVG), D2 deletion (ΔD2, missing amino acids 1251–1523, AQVE…EYLG), D2D2 (amino acids 993–1232 replaced by amino acids 1251–1523), PPLL, QFG, and EGFID were generated based on C1-YFP-mouse PTPσ with four FNIII domains and lacking both the meA and meB splice inserts (Takahashi et al., 2011). The V5 tag was inserted directly after the signal peptide and YFP was removed, and the WT construct (which was used as the template to construct all mutants) was made shRNA-resistant by mutating the sequence GGCATCATGGGTAGTGATT to GGaATaATGGGaAGcGATT.

C1-myc-trio (Terry-Lorenzo et al., 2016) was a kind gift from Dr Craig Garner (German Center for Neurodegenerative Diseases, Bonn, Germany) and contains the human trio cDNA sequence. Mouse caskin1 cDNA (accession number BC060720) was obtained from Open Biosystems. A small portion of the 5′ end of the cDNA was missing and was restored by PCR.

CMV-HA-liprin-α2 containing the mouse liprin-α2 gene was a kind gift from Dr. Susanne Schoch (Institute of Neuropathology, Bonn, Germany) containing the mouse liprin-α2 gene. We mutated the sequence GGGGCTGATCCACCGGAGTTT to GGaGCcGATCCtCCaGAaTTT in order to make the sequence resistant to an shRNA (not used in this work) without changing the amino acid sequence. From the resulting plasmid, we transferred the open reading frame to the pBA vector, which contains the CAG chicken β-actin promoter and was a kind gift from Dr Gary Banker (Oregon Health and Sciences University, Portland, OR, USA), and replaced the N-terminal HA tag with a myc tag (EQKLISEEDL) to generate pBA-myc-liprin-α2.

Fusions with the dihydrofolate reductase (DHFR) F3 C-terminal fragment were made based on p41-HPH-TEF-SspBYGMF-linker-DHFR-F3 (Tarassov et al., 2008). PTPσ, liprin-α2, caskin1, and trio sequences were subcloned either in full or in truncated form from the plasmids described above so that they were fused at their C-termini *via* a short linker to the F3 fragment. PTPσ ICR consisted of amino acids 974–1530 (KLSQ…HYAT), liprin-α2 SAM consisted of amino acids 877–1192 (KDRR…SDDK), caskin1 SAM consisted of amino acids 344–636 (AIVK…MAIE), and trio IgPSK consisted of amino acids 2237–3062 (NQRN…LPRV). Fusions with the DHFR F1–2 N-terminal fragment were cloned into the p413 vector (Mumberg et al., 1995). A cassette containing the DHFR F1–2 fragment and the Adh1 terminator from pAG25-F1–2 (Tarassov et al., 2008) was fused to the C-terminus of PTPσ full-length (FL) or ICR under the control of the TEF promoter. PTPσ-FL-DHFR both F1–2 and F3 contained an N-terminal V5 tag. NgCAM, which was a kind gift from Dr Peter Sonderegger (University of Zurich, Zurich, Switzerland), and YFP were each separately fused to F1–2 and to F3 as controls. Point mutations were introduced into PTPσ FL or ICR DHFR F1–2 fusions, and also into the PTPσ FL DHFR F3 fusion in the case of the PPLL mutant (Hofmeyer and Treisman, 2009).

### Neuron Culture, Transfection, and AAV Transduction

This study was carried out in accordance with the recommendations of the Canadian Council on Animal Care. The protocol was approved by the University of British Columbia Animal Care Committee.

Primary embryonic day 18 (E18) rat hippocampal neurons were cultured essentially according to the method described in Kaech and Banker (2006). For expression of DNA constructs, the AMAXA nucleofector system (Lonza) was used to deliver an appropriate amount of plasmid (2–4 μg per construct) to 1–2 million freshly dissociated cells. Cells were plated on poly-L-lysine-coated coverslips at an initial density of approximately 700,000–900,000 for transfected neurons, or 500,000 for untransfected neurons, per 6-cm dish. Neurons were maintained with a glial feeder layer, and cytosine arabinoside (5 μM) was added at DIV 2 to prevent glial overgrowth. DL-2-amino-5-phosphonovaleric acid (APV, 100 μM) was added starting from DIV 5 in order to limit excitotoxicity and improve neuronal survival.

Delivery of AAVs was accomplished by incubating DIV 6 neurons face-up in 12-well plates, each well containing 700 μL of glial-conditioned media with 5 × 10^9^ viral genomes (vg) of the appropriate viral construct. Conditioned media was harvested from either the neurons’ own dishes or from similar dishes and was centrifuged for 5 min at 1,500 × *g* before use. APV was added at a concentration of 100 μM. Neurons were incubated in the virus-containing solution for 4 h and then transferred back to their home dishes.

### Real Time Quantitative PCR (RT-qPCR)

RT-qPCR was performed to confirm triple knockdown of PTPσ, PTPδ, and LAR by AAV6-shPTP. Neurons were treated with AAV carrying shCtrl or shPTP as above, harvested on DIV 16 in cold PBS, and then lysed in TRIzol reagent (Invitrogen). RNA extraction from lysates was performed immediately using the PureLink RNA Mini Kit (Thermo Fisher Scientific). Elimination of genomic DNA and retro-transcription to cDNA were performed using SuperScript IV Vilo Master Mix with ezDNase Enzyme (Thermo Fisher Scientific). SYBRGreen qPCR (PowerUp^TM^ SYBR Green Master Mix, Thermo Fisher Scientific) was performed using the resulting cDNA, with glyceraldehyde-3-phosphate dehydrogenase (GAPDH) and β-actin (Actb) as reference genes. Primers used are listed in the table below, and their efficiency was estimated as between 0.87 and 1.05. All quantitation cycle (Cq) values were detected prior to completion of the 38th cycle.

**Table:** List of primers used in quantitative RT-PCR assays.

Gene (Rat)	Forward Primer 5′–3′	Reverse Primer 3′–5′
PTPσ	CTTGAGTTCAAGAGGCTTGC	GTCTGTAGCCGTCGATGAAG
PTPδ	CCATGCAGAGTCCAAGATGT	GACAGGACCTACGACCCATA
LAR	CTTCAAGCTCTCTGTTCACTGC	ACCCCGCCTAATGTATAAACG
GAPDH	AGACAAGATGGTGAAGGTCG	TCGTTGATGGCAACAATGTC
Actb	GATCAAGATCATTGCTCCTCCTG	AAGGGTGTAAAACGCAGCTC

### Knockdown Confirmation by Western Blot

Neurons were treated with AAV carrying shCtrl or shPTP as above and harvested for Western blotting at DIV 17–18 using Complexiolyte-48 (50 μL/coverslip, Logopharm). Protein concentrations were determined, and equal amounts were run on 10% SDS-polyacrylamide gels. Proteins were blotted using Immobilon P membranes (Millipore). Membranes were blocked using 5% skim milk in Tris-buffered saline with 0.001% Tween-20 and incubated in antibody solution. Primary antibodies used were anti-PTPσ (mouse, 17G7.2, MM-0020, Medimabs) and anti-β-actin (rabbit, 1:5,000, ab8227, Abcam). Secondary antibodies were goat anti-mouse or goat anti-rabbit HRP conjugate from Southern Biotech. Detection was performed using Immobilon Western Chemiluminescent substrate (Millipore).

### Coculture Assay

COS cells were maintained in Dulbecco’s modified Eagle’s medium (DMEM) with 10% bovine growth serum (BGS). When neurons reached the age of DIV 12, near-confluent COS cells were harvested using 0.25% trypsin-EDTA and plated in 12-well plates, then transfected the following day at ~85% confluence using polyethylenimine (Boussif et al., 1995) with 1 μg of plasmid DNA encoding a tagged form of either a postsynaptic organizing protein or CD4 as a non-synaptogenic control. The day after transfection, when neurons were at DIV 13, COS cells were harvested again as before, washed twice with DMEM with 10% BGS, resuspended in glial conditioned media (treated as above for viral infection), and plated at a density of ~20,000 cells per coverslip on top of the neurons. Cocultures were allowed to incubate for 18–24 h prior to fixation and staining.

### HEK Cell Cultures and Clustering Assay

Maintenance, harvesting, and transfection of HEK 293 (HEK) cells were performed in the same manner as for COS cells, except that the concentration of trypsin-EDTA used was 0.05%. Prior to transfection, cells were plated in 12-well plates on coverslips coated in poly-D-lysine (PDL). The clustering assay was performed by transfecting HEK cells with a mixture of 0.2 μg of pBA-myc-liprin-α2, 0.6 μg of pLL-V5-PTPσ WT or mutant, and 0.2 μg of pLL CFP. Control coverslips replaced either the liprin-α2 or the PTPσ plasmid with an equal amount of additional pLL CFP plasmid, such that the total DNA amount was always 1 μg.

### Immunocytochemistry and Imaging

Live surface staining for both HEK cells (Figure 7) and cocultures (Figures 2–4) was performed by incubating coverslips face-up in antibody solution for 30 min on an ice block in a 37°C, 5% CO_2_, humidified incubator. Antibody solution was made with fresh Neurobasal media. APV was added at a concentration of 100 μM for experiments involving neurons. Coverslips were washed with Neurobasal media three times prior to fixation.

**Figure 1:**
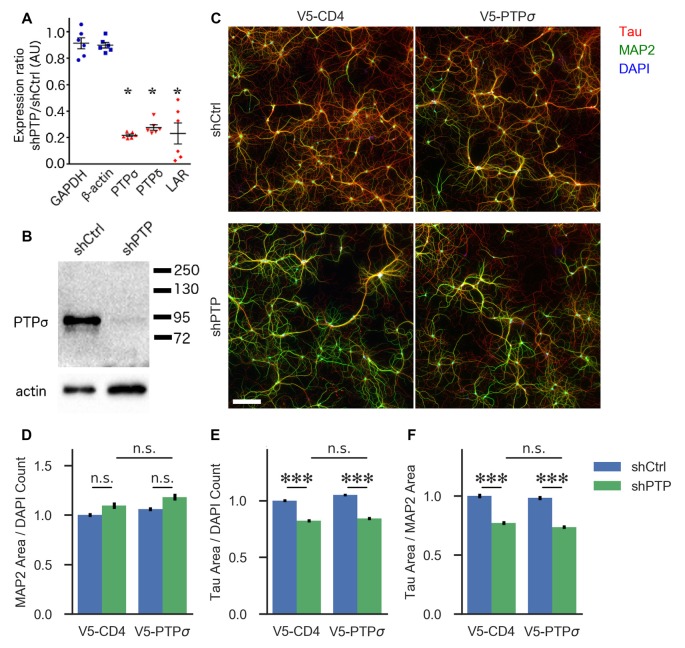
Knockdown confirmation and morphological characterization of neurons with reduced levels of LAR-RPTPs.** (A)** Quantification of mRNA levels for the three LAR-RPTPs, as well as glyceraldehyde-3-phosphate dehydrogenase (GAPDH) and β-actin as control reference genes, measured by RT-qPCR in hippocampal neurons treated with AAV shPTP relative to shCtrl. *p < 0.0001 vs. GAPDH, ANOVA with *post hoc* Dunnett’s multiple comparison test, *n* = 3 biological and three technical replicates each from two independent cultures (each point represents one biological replicate). AAVs were applied at DIV 6, and neurons were harvested at DIV 16. **(B)** Western blot against protein tyrosine phosphatase σ (PTPσ) in hippocampal neurons treated with either shCtrl or shPTP. AAVs were applied at DIV 6, and neurons were harvested at DIV 16. The full-size blot is shown in Supplementary Figure S1. **(C)** Example images of hippocampal neurons electroporated with either V5-CD4 or V5-PTPσ (columns) and treated at DIV 6 with AAVs carrying either shCtrl or shPTP sequences (rows). Neurons were fixed at DIV 14, stained for Tau (axons), MAP2 (dendrites), and DAPI (nuclei), and imaged. Scale bar represents 200 μm. **(D)** Quantification of total MAP2-positive area per field, normalized to the number of DAPI-stained nuclei in the field. Overall *p*-value for the experiment was 0.00157, Kruskal-Wallis, *n* = 160–161 fields per condition from four independent cultures. n.s., not significant, *p* > 0.05 by Dunn’s *post hoc* test. **(E)** Quantification of total Tau-positive area per field, normalized to the number of DAPI-stained nuclei in the same field. Overall *p*-value for the experiment was 2.021 × 10^−36^, Kruskal-Wallis. ***p < 0.001, Dunn’s *post hoc* test, n.s., not significant. **(F)** Quantification of the ratio between Tau and MAP2 total area per field. Overall *p*-value for the experiment was 1.578 × 10^−36^, Kruskal-Wallis. ***p < 0.001, Dunn’s *post hoc* test, n.s., not significant.

**Figure 2:**
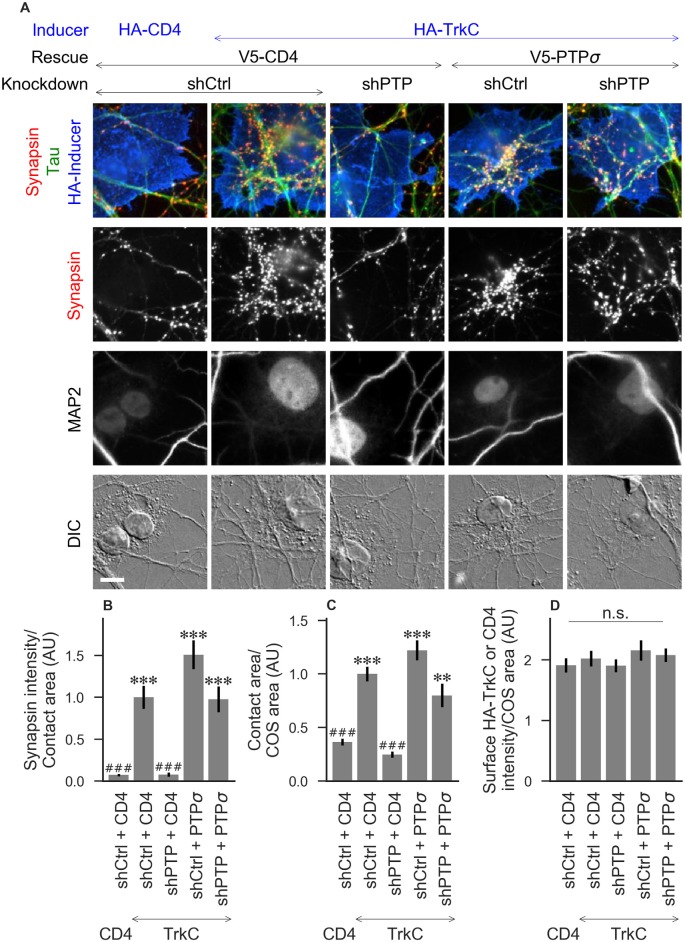
Synaptogenic activity of TrkC is abolished by LAR-RPTP triple knockdown and rescued by PTPσ. **(A)** Representative images of HA-TrkC cocultures in which neurons were treated with either control (shCtrl) or LAR-RPTP triple knockdown (shPTP)-expressing AAVs, and rescued using V5-CD4 as a control or RNAi-resistant V5-PTPσ. Left-most column shows neurons cocultured with COS cells expressing HA-CD4 as a negative control. Rescue constructs were introduced by nucleofection at DIV 0, AAV shRNAs were applied at DIV 6, and coculture assays were performed at DIV 13–14. Synapsin was recruited in tau-positive axons at sites of contact with TrkC-expressing (but not CD4-expressing) COS cells. Microtubule-associated protein 2 (MAP2) labeling of dendrites was used to exclude native synapses from analysis. Scale bar represents 10 μm. **(B–D)** Quantification of synapsin recruitment, contact area between tau-positive axons and transfected COS cells, and intensity of HA-CD4 or HA-TrkC on the COS cell surface, from the experiment shown in **(A)**. “Contact area” is defined as the region where axons contact the inducer-expressing COS cell, excluding the area overlapped by MAP2-positive dendrites. “COS area” also excludes areas of MAP2 overlap. Values are normalized to the mean value in the shCtrl + CD4 with TrkC coculture condition from the same culture. Data are expressed as mean ± SEM. Overall *p*-values were 5.05 × 10^−20^ **(B)**, 4.02 × 10^−19^ **(C)**, and 0.66 **(D)**, Kruskal-Wallis, *n* = 28–32 cells per condition from three independent cultures. ***p < 0.001, **p < 0.01 compared to shCtrl + CD4 with CD4 coculture, ^###^*p* < 0.001 compared to shCtrl + CD4 with TrkC coculture, Dunn’s *post hoc* test, n. s., not significant.

**Figure 3:**
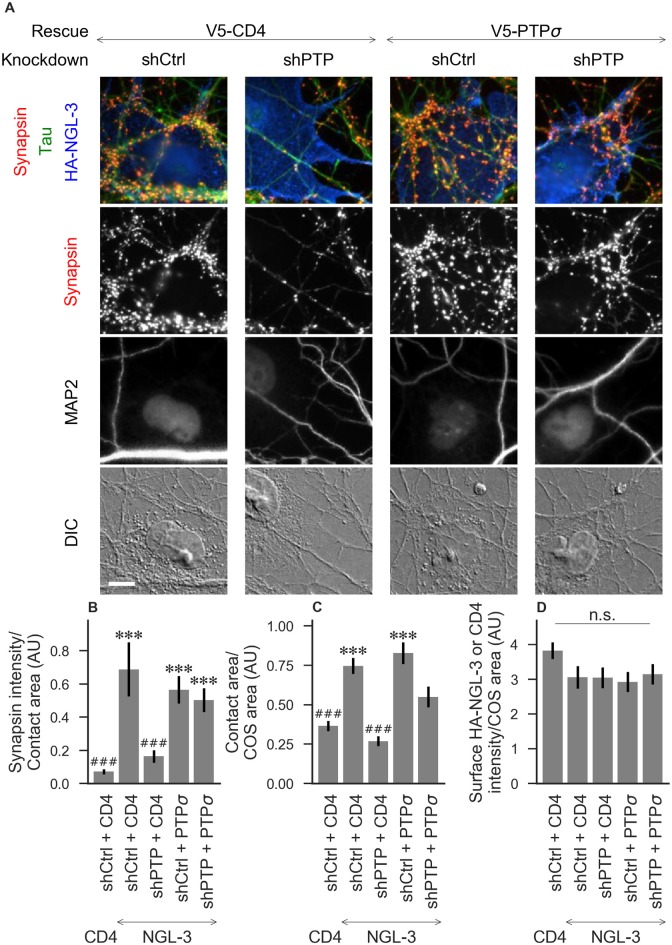
Synaptogenic activity of NGL-3 is abolished by LAR-RPTP triple knockdown and rescued by PTPσ. **(A)** Representative images of HA-NGL-3 cocultures in which neurons were treated with either control (shCtrl) or LAR-RPTP triple knockdown (shPTP)-expressing AAVs, and rescued using V5-CD4 as a control or RNAi-resistant V5-PTPσ. Rescue constructs were introduced by nucleofection at DIV 0, AAV shRNAs were applied at DIV 6, and coculture assays were performed at DIV 13–14. Synapsin was recruited in tau-positive axons at sites of contact with NGL-3-expressing COS cells. MAP2 labeling of dendrites was used to exclude native synapses from analysis. Scale bar represents 10 μm. **(B–D)** Quantification of synapsin recruitment, contact area between tau-positive axons and transfected COS cells, and intensity of HA-CD4 or HA-NGL-3 on the COS cell surface, from the experiment shown in **(A)**. “Contact area” is defined as the region where axons contact the inducer-expressing COS cell, excluding the area overlapped by MAP2-positive dendrites. “COS area” also excludes areas of MAP2 overlap. Values are normalized to the mean value in the shCtrl + CD4 with TrkC coculture condition from the same culture. Data are expressed as mean ± SEM. Overall *p*-values were 6.69 × 10^−13^ **(B)**, 4.07 × 10^−13^ **(C)**, and 0.034 (**D**, although none of the individual comparisons were significant based on Dunn’s *post hoc* test), Kruskal-Wallis, *n* = 27–31 cells per condition from three independent cultures. ***p < 0.001 compared to shCtrl + CD4 with CD4 coculture, ^###^*p* < 0.001 compared to shCtrl + CD4 with NGL-3 coculture, Dunn’s *post hoc* test. CD4 coculture condition is the same as that shown in Figure 2 (see Figure 2A for example image), n. s., not significant.

**Figure 4:**
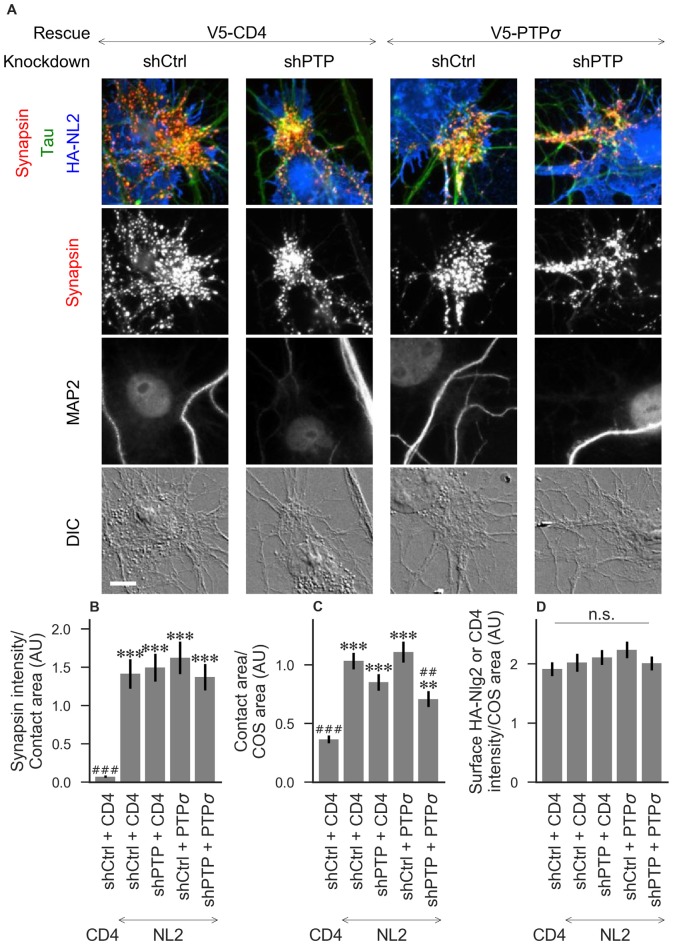
Synaptogenic activity of neuroligin-2 (NL2) is unaffected by LAR-RPTP triple knockdown or expression of PTPσ. **(A)** Representative images of HA-NL2 cocultures in which neurons were treated with either control (shCtrl) or LAR-RPTP triple knockdown (shPTP)-expressing AAVs, and rescued using V5-CD4 as a control or RNAi-resistant V5-PTPσ. Rescue constructs were introduced by nucleofection at DIV 0, AAV shRNAs were applied at DIV 6, and coculture assays were performed at DIV 13–14. Synapsin was recruited in tau-positive axons at sites of contact with NL2-expressing COS cells. MAP2 labeling of dendrites was used to exclude native synapses from analysis. Scale bar represents 10 μm. **(B–D)** Quantification of synapsin recruitment, contact area between tau-positive axons and transfected COS cells, and intensity of HA-CD4 or HA-NL2 on the COS cell surface, from the experiment shown in **(A)**. “Contact area” is defined as the region where axons contact the inducer-expressing COS cell, excluding the area overlapped by MAP2-positive dendrites. “COS area” also excludes areas of MAP2 overlap. Values are normalized to the mean value in the shCtrl + CD4 with TrkC coculture condition from the same culture. Data are expressed as mean ± SEM. Overall *p*-values were 4.70 × 10^−15^ **(B)**, 2.44 × 10^−13^ **(C)**, and 0.40 **(D)**, Kruskal-Wallis, *n* = 26–32 cells per condition from three independent cultures. ***p < 0.001, **p < 0.01 compared to shCtrl + CD4 with CD4 coculture, ^###^*p* < 0.001, ^##^*p* < 0.01 compared to shCtrl + CD4 with NL2 coculture, Dunn’s *post hoc* test. CD4 coculture condition is the same as that shown in Figure 2 (see Figure 2A for example image), n. s., not significant.

**Figure 5:**
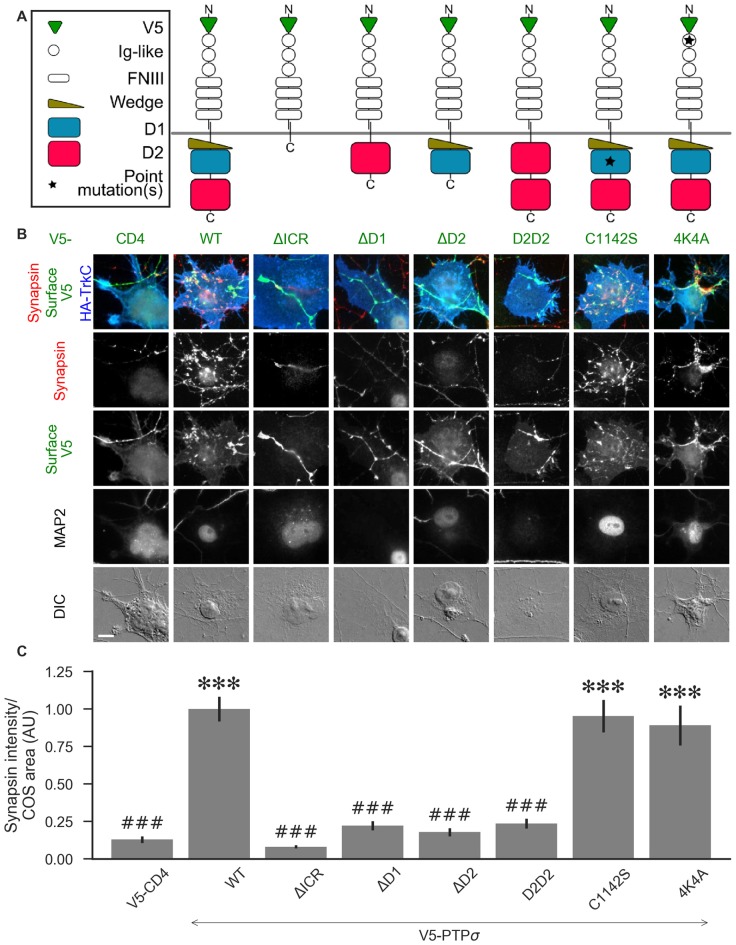
PTPσ requires its D1 and D2 domains, but not its phosphatase activity or heparan sulfate (HS)-binding, to mediate TrkC synaptogenic activity. **(A)** Schematic view of PTPσ mutants used in this experiment. Each schematic corresponds to the label and column of images directly below it in **(B)**. **(B)** Representative images of HA-TrkC cocultures where neurons were treated with shPTP-expressing AAVs, and rescued using V5-CD4 as a control or RNAi-resistant V5-PTPσ carrying the indicated mutations. Rescue constructs were introduced by nucleofection at DIV 0, AAV shRNAs were applied at DIV 6, and coculture assays were performed at DIV 13–14. MAP2 labeling of dendrites was used to exclude native synapses from analysis. Scale bar represents 10 μm. **(C)** Quantification of synapsin recruitment shown in **(B)**. “COS area” indicates only the portion of the TrkC-expressing COS cell not overlapping with MAP2 signal. All values are normalized to the mean of the wild-type (WT) condition from the same culture. Data are expressed as mean ± SEM. Overall *p*-value for this experiment was 1.32 × 10^−44^, Kruskal-Wallis, *n* = 33–100 cells per condition from 2–3 cultures. ***p < 0.001 compared to CD4, ^###^*p* < 0.001 compared to WT, Dunn’s *post hoc* test. There were no differences in surface levels of the V5-PTPσ mutants (Supplementary Figure S2A).

**Figure 6:**
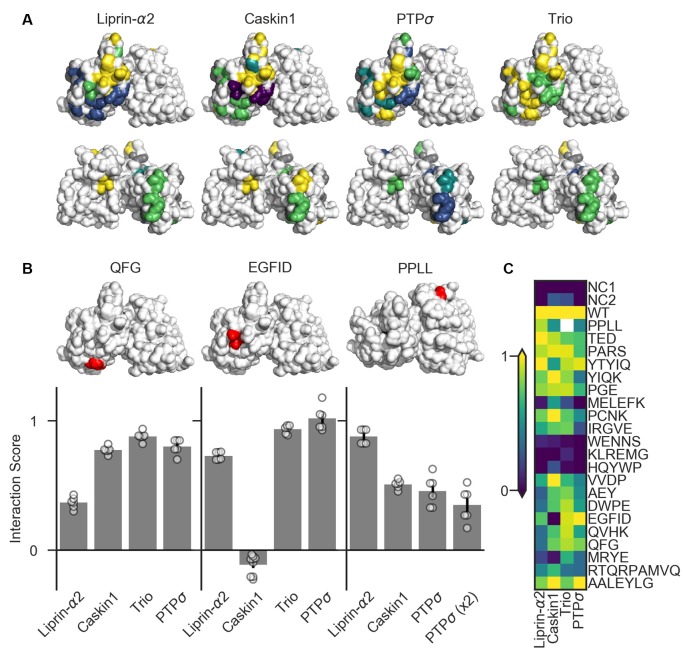
Identification of PTPσ mutations to disrupt specific interactions. **(A)** Interaction scores for each ligand in the dihydrofolate reductase (DHFR) protein complementation assay mapped onto the PTPσ crystal structure (Hou et al., 2011). Interaction scores are divided into five bins and colored accordingly: <0.2 purple; 0.2–0.4 blue; 0.4–0.6 teal; 0.6–0.8 green; >0.8 yellow. Mutants which showed an interaction score <0.3 for at least three ligands are shown in gray. Top row shows the D1 domain on the right and D2 on the left. Second row shows the same structures, flipped horizontally. **(B)** Quantification of mutants that were used in subsequent experiments. Models above each plot are the same crystal structures as shown in the top row in **(A)**, with the indicated mutation shown in red. The model for PPLL is rotated forward slightly relative to the others in order to make these residues visible. Note that horizontal axis is different for PPLL since this mutation was not tested for interaction with trio, and since in the case of PTPσ homodimerization we also tested the condition where both copies of PTPσ carried the mutation [PTPσ (x2)]. Data are mean ± SEM. **(C)** Heatmap showing interaction scores for 22 different PTPσ multi-point mutations. NC1 indicates the negative control where the DHFR C-terminal fragment was fused to YFP or NgCAM instead of the indicated ligand; NC2 indicates the negative control where the DHFR N-terminal fragment was fused to NgCAM or YFP instead of to PTPσ. Interaction scores represent the growth rate of the indicated strain in MTX-containing media relative to MTX-free media and relative to controls, with 0 indicating the same relative growth rate as NC1 and one indicating the same relative growth rate as WT PTPσ. Values higher than 1 or lower than 0 are clipped to 1 and 0. White indicates no data (residues affected by the PPLL mutation are not present in the fragment of PTPσ used to test interaction with trio). Data are based on two experiments per condition and three replicates per experiment.

**Figure 7:**
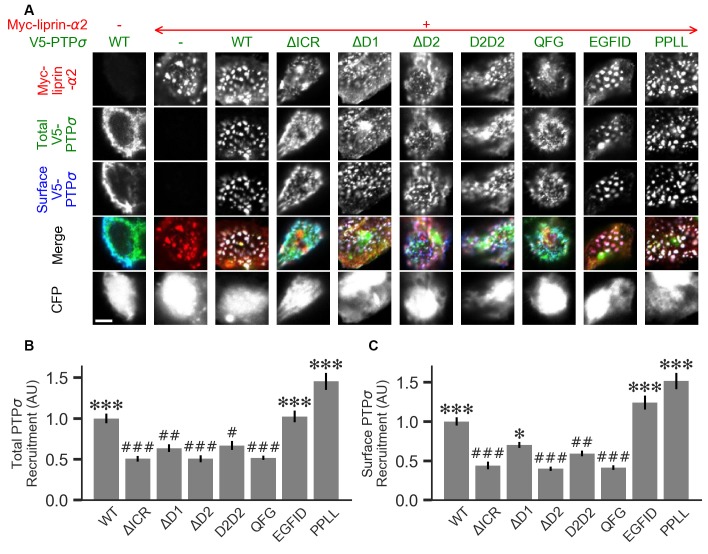
Measurement of PTPσ/liprin-α2 interaction based on co-clustering in HEK cells. **(A)** Representative images of HEK cells transfected with myc-liprin-α2, V5-PTPσ WT or mutant, and CFP. Scale bar represents 5 μm. **(B)** Quantification of the extent to which PTPσ was recruited to sites of liprin-α clustering. “Total PTPσ Recruitment” refers to the ratio between the intensity of V5-PTPσ colocalized with myc-liprin-α2 puncta vs. the remaining area of the cell, normalized to the mean value for cells in the WT condition from the same culture. Overall *p*-value was 7.5 × 10^−23^, Kruskal-Wallis. *p < 0.05, ***p < 0.001 compared to WT, ^#^*p* < 0.05, ^##^*p* < 0.01, ^###^*p* < 0.001 compared to intracellular region (ΔICR), Dunn’s *post hoc* test. **(C)** Same as **(B)**, but using surface stained V5-PTPσ rather than total. Overall *p*-value was 2.3 × 10^−31^, Kruskal-Wallis. *p < 0.05, ***p < 0.001 compared to WT, ^#^*p* < 0.05, ^##^*p* < 0.01, ^###^*p* < 0.001 intracellular region (ΔICR), Dunn’s *post hoc* test.

Cells were fixed in warm phosphate-buffered saline (PBS) with 4% paraformaldehyde and 4% sucrose for 12 min. Surface staining of V5-PTPσ in neurons was performed after fixation but before permeabilization. For experiments involving surface staining of both V5-PTPσ and HA, staining was performed after fixation for both antibodies. Non-permeabilized cells were blocked for 30 min in blocking buffer (3% BSA, 5% normal goat serum in PBS) at 37°C, then incubated in primary antibody solution in blocking buffer at 4°C for two nights prior to permeabilization. Cultures were permeabilized using 0.2% Triton-X100 in PBS for 5 min. Cultures were blocked as above and remaining primary antibodies were applied in blocking buffer overnight at 4°C. Secondary antibodies were applied in blocking buffer for 45 min at 37°C.

Primary antibodies used were mouse IgG1 anti-SynapsinI (Synaptic Systems, cat no. 106011, 1:40,000, used in Figures 2–4), rabbit anti-SynapsinI (Millipore, cat no. ab5905, 1:10,000, used for all other synapsin staining), mouse IgG2a anti-TauI (Millipore, cat no. PC1C6, 1:2,000), chicken anti-microtubule-associated protein 2 (anti-MAP2; Abcam, cat no. ab5392, 1:2,000), mouse IgG2b anti-HA (Abcam, cat no. 12CA5, 1:500), mouse IgG1 anti-myc (Santa Cruz, cat no. sc-40, 1:500, used in Figure 7) rabbit anti-myc (Sigma, cat no. C3956, 1:2,000, used for all other myc staining), rabbit anti-V5 (Cell Signalling Technology, cat no. 13202, 1:5,000, used in Figure 7 for total V5 staining), and mouse IgG2a anti-V5 (Thermo Fisher, cat no. R960, 1:5,000, used for all V5 surface staining). Secondary antibody against chicken was AMCA AffiniPure Goat Anti-Chicken IgY (IgG; H + L; Jackson, cat no. 103155, 1:200). All other secondary antibodies were from Thermo Fisher and generated in goat, conjugated to Alexa Fluor 488, 568, or 647. Alexa Fluor-conjugated secondary antibodies were used at a concentration of 1:1,000.

Imaging of HEK cells for the co-clustering assay was performed on a Zeiss LSM700 with a 40×/1.4 NA oil immersion objective at 2× zoom, using single optical sections. Cells were selected based on the channels containing CFP and liprin-α2 only. Cells without expression of both CFP and liprin-α, as well as cells showing diffuse liprin-α signal, were avoided.

For all other experiments, imaging was performed on either the same microscope in epifluorescence mode or on an Axioplan 2, with either a 10×/0.45 NA air (morphology experiments shown in Figure 1) or a 40×/1.4 NA oil immersion (all others) objective and a Hamamatsu Orca-Flash4.0 CMOS camera. For experiments imaged using the LSM700, images were acquired as a z-stack containing three slices at 1.46 μm spacing (10×), or 11 slices at 0.23 μm spacing (40×), then combined into a single plane using the Zeiss Extended Depth of Focus module. All imaging was performed blind to experimental condition.

### Quantification, Statistical Analysis, and Data Visualization

The order of images was randomized, and measures were performed blind to experimental condition. Images were analyzed in FIJI (NIH) using custom-written Python scripts. Regions of interest (ROIs) were generated by manually choosing a lower brightness threshold for each image and then converting the thresholded image to an ROI using the command “Create Selection.” For experiments where measurements were taken from an ROI based on multiple channels, single-channel ROIs were combined using the ROI Manager tool. For coculture experiments, the ROI based on the MAP2 channel was first dilated by five pixels using the command “Enlarge,” then inverted using “Make Inverse.” The purpose of these two steps was to limit the final ROI to areas of the image which did not contain dendrites and to also exclude the area immediately around the dendrite which could contain spine-associated synapses not directly overlapping the MAP2 signal. In the coculture assay, the threshold for synapsin or myc-liprin-α2 was chosen to include only punctate signal. Thus, the final measure was punctate synapsin or myc-liprin-α2 per COS cell area or per COS cell axon contact area, all lacking dendrite contact. For the HEK cell co-clustering assay, a background subtraction step was first performed on the myc-liprin-α2 channel using the rolling ball method with a radius of 14 pixels. ROIs were generated by thresholding the CFP cell-fill and background-subtracted myc-liprin-α2 channels. Combined ROIs representing CFP-positive, myc-liprin-α2-positive; and CFP-positive, myc-liprin-α2-negative regions were used to measure the surface and total V5-PTPσ channels. The V5-PTPσ channels were not thresholded. The mean intensity within the first ROI divided by that within the second was used as a measure of the strength of co-clustering.

For experiments in which cells were chosen in order to ensure even surface expression of V5 across conditions, cells showing high or low V5 levels were excluded before analyzing downstream measures such as synapsin intensity.

Analysis of image measurement results and DHFR assay growth data, statistical analysis, and data visualization were performed using Jupyter Notebook (Kluyver et al., 2016), Python 3, and the following packages: pandas, numpy, re, scipy.stats, scikit_posthocs, seaborn, and matplotlib. Data were tested for normality using scipy.stats.normaltest, and analyzed using non-parametric tests as indicated in figure legends if found to be non-normal. Visualization of protein crystal structures was performed using the PyMOL Molecular Graphics System, Version 1.8.6.0 Schrödinger, LLC.

### Yeast Cultures and DHFR Assay

Yeast (strain BY4741) were transformed with two plasmids, the first containing DHFR-F1–2 fused to the C-terminus of either PTPσ FL or ICR or a control, and the second containing DHFR-F3 fused to the C-terminus of the indicated interacting protein or a control. DHFR-F3 constructs contained the SAM domains of liprin-α2 and caskin1, the immunoglobulin-like and protein serine kinase domains (IgPSK) of trio, or the FL version of PTPσ. PTPσ ICR-DHFR-F1–2 was used to test for interactions with trio IgPSK since preliminary experiments (not shown) revealed that the growth rate of yeast transformed with trio IgPSK and PTPσ FL was very low. All other DHFR-F3 constructs were transformed along with PTPσ FL-DHFR-F1–2. Negative controls replaced either the F1–2 or the F3 fusion with a control protein of a similar size. PTPσ FL was replaced by NgCAM. PTPσ ICR, liprin-α2 SAM, caskin1 SAM, and trio IgPSK were all replaced by YFP. Transformed strains were grown on synthetic defined (SD) media lacking histidine and containing hygromycin (100 μg/mL) to select for doubly transformed cells.

Transformed strains were grown in SD media lacking histidine and adenine (Michnick et al., 2016) and containing hygromycin overnight at 30°C with shaking, then diluted in a 96-well plate with 200 μL/well to an OD of 0.05 in the same media containing 1% DMSO with or without 200 μg/mL methotrexate (MTX). Each plate contained a positive control strain with two WT interacting proteins and two YFP or NgCAM negative controls. Each yeast strain had three replicate wells each with DMSO alone or DMSO + MTX. The plate lid was coated with TritonX-100 (0.05% in 20% ethanol) to minimize condensation, and the cultures were incubated in a BioTek Epoch 2 plate reader at 29–31°C (2° gradient intended to prevent condensation on the plate lid) with OD_600_ readings taken every 10 min at least until the blanked log_2_(OD_600_) of all samples reached a value of −2, which generally took between 20 and 30 h.

Data were inspected visually for any wells showing abrupt drops in OD_600_ readings or other abnormalities, and such wells were excluded from the dataset. Data were log_2_ transformed. For each well, the slope of the growth curve in the linear range between log_2_(OD_600_) values of −3 and −2 (or between −3 and −2.2 in the case of one experiment) was calculated. For each MTX-containing well, the ratio between that well’s slope value and the mean slope value for DMSO-containing wells from the same experiment containing the same strain was calculated. Interaction scores were calculated according to the formula (ratio_x_ − ratio_NC_)/(ratio_PC_ − ratio_NC_), where ratio_x_, ratio_NC_, and ratio_PC_ are the MTX/DMSO ratios of the strain of interest, the negative control containing either NgCAM-DHFR-F3 or YFP-DHFR-F3, and the positive control which was PTPσ WT-F1–2 co-transfected with the appropriate WT interacting protein-F3, respectively.

## Results

### Role of LAR-RPTPs in Neuronal Morphology and Presynaptic Differentiation

First, we assessed the role of the LAR-RPTP family in regulating neuronal morphology in cultured hippocampal neurons. Although this aspect of LAR-RPTP function has been extensively studied in other contexts (Ledig et al., 1999; McLean et al., 2002; Thompson et al., 2003; Chagnon et al., 2004; Sapieha et al., 2005; Siu et al., 2007; Horn et al., 2012; Stoker, 2015) and was not a primary focus of this work, understanding the effects of LAR-RPTP knockdown on outgrowth in our system was necessary for designing experiments focused on their role in synapse formation. To this end, we employed an AAV carrying shRNAs targeting PTPσ, PTPδ, and LAR (shPTP) or carrying four copies of an shRNA targeting GFP (shCtrl) as a control. Infection of primary hippocampal neurons with shPTP reduced levels of all three LAR-RPTPs by 70%–80% compared to shCtrl as assessed by RT-qPCR (Figure 1A), and strongly reduced levels of PTPσ as assessed by Western blot (Figure 1B). Neurons were fixed at DIV 14 and stained with antibodies against Tau and MAP2 to mark axons and dendrites, respectively (example images shown in Figure 1C). We observed a consistent reduction in the area occupied by Tau-positive axons, either alone or as a ratio with MAP2-positive dendrite area, in response to shPTP (Figures 1E,F). In order to test the ability of PTPσ to rescue this defect, we transfected neurons with either shRNA-resistant V5-PTPσ or V5-CD4 as a control prior to infection with shCtrl or shPTP. However, this manipulation was not able to rescue the outgrowth phenotype (Figures 1E,F; see “Discussion” section). Thus, LAR-RPTPs were required for normal axon outgrowth.

To assess the role of LAR-RPTPs in presynaptic differentiation, we used a neuron-fibroblast coculture assay. When COS cells are transfected with the appropriate postsynaptic organizing protein and then added to primary neuronal cultures, presynaptic differentiation is induced at contact sites by local aggregation of axonal LAR-RPTPs or neurexins (Scheiffele et al., 2000; Takahashi and Craig, 2013). The coculture assay allowed us to control for differences in axon growth by normalizing recruitment of the presynaptic marker synapsin to the axon contact area. Further, in the coculture assay, we could isolate presynaptic differentiation induced by LAR-RPTP ligands TrkC and NGL-3 from differentiation induced by neurexin ligands such as neuroligin-2 (NL2). We cocultured neurons treated as above with COS cells expressing HA-tagged CD4, TrkC, NGL-3, or NL2 from DIV 13–14 (example images shown in Figure 2A for CD4 and TrkC, Figure 3A for NGL-3, and Figure 4A for NL2). As expected, the non-synaptogenic molecule CD4 did not induce clustering of synapsin. Under control conditions (V5-CD4 and shCtrl), TrkC induced robust recruitment of synapsin reflecting presynaptic differentiation at sites of contact between the axons and COS cells, and this was abolished by treatment with shPTP. Cultures transfected with V5-PTPσ showed synapsin recruitment when treated with either shCtrl or shPTP, indicating that PTPσ was able to rescue the synaptogenic activity of TrkC (Figure 2B). These measures of synapsin recruitment were normalized to axon contact area, reflecting local presynaptic differentiation. Measuring the extent to which axons were recruited to transfected COS cells revealed the same broad trend in that axon recruitment to TrkC-expressing COS cells was reduced by shPTP and largely rescued by expression of V5-PTPσ (Figure 2C). Differences in recruitment of axons and in synapsin clustering could not be explained by differences in surface levels of TrkC between conditions (Figure 2D). NGL-3 showed similar results as TrkC, although the ability of PTPσ to rescue axon recruitment was somewhat weaker in this case (Figures 3B–D). Neurons treated with shPTP + V5-PTPσ and cocultured with NGL-3 did not show significant differences in axon recruitment from either the positive (shCtrl + V5-CD4, cocultured with NGL-3) or the negative (shCtrl + V5-CD4, cocultured with CD4) control. This partial rescue is consistent with NGL-3’s dependence on other ligands within the LAR-RPTP family (Kwon et al., 2010). Synaptogenic activity of NL2 as measured by recruitment of synapsin was unaffected by shPTP. There was a slight reduction in axon recruitment by NL2 in shPTP-treated neurons, which was significant compared to the positive control (shCtrl + V5-CD4, cocultured with NL2) in the shPTP + V5-PTPσ condition, consistent with an overall reduction in axon outgrowth as a result of knockdown of LAR-RPTPs (Figures 4B–D).

### Domain Analysis of PTPσ

We next sought to understand which domains, molecular interaction sites, and enzymatic activities of PTPσ were required for its synaptogenic activity. To do this, we used a molecular replacement strategy in which neurons were treated with shPTP-containing AAV to knock down native PTPσ and rescued with V5-tagged WT or mutant PTPσ, or CD4 as a negative control. In this and all other experiments involving transfection in neurons, shRNA-resistant versions of PTPσ (either WT or mutant) were used. Since the synaptogenic activity of TrkC was abolished by LAR-RPTP knockdown and fully rescued by PTPσ, for this and all subsequent assays to dissect mechanisms of PTPσ-mediated presynaptic differentiation, we used TrkC cocultures.

We hypothesized that, because liprin-α, caskin, and trio all bind to the D2 domain, while there are no known binding partners of the D1 domain aside from PTPσ itself, loss of the D2 domain would prevent PTPσ from mediating presynaptic differentiation in the coculture assay. We also thought it possible that phosphatase activity against p250GAP, β-catenin, N-cadherin, or an unknown target could be necessary for PTPσ’s synaptogenic activity, meaning that the D1 domain would also be required. Alternatively or in addition, the D1 domain could act as a spacer between the cell membrane and the D2 domain thus allowing D2 to assemble a multi-protein presynaptic complex. Replacing the D1 domain with a second copy of D2 would restore such a steric role for the D1 domain but not a specific catalytic or interaction role. We further asked whether HSPG binding to a co-receptor might be needed for PTPσ’s synaptogenic activity. Thus, we tested the following mutants: a deletion of the entire intracellular region (ΔICR), a deletion of the D1 or D2 domain (ΔD1 and ΔD2), a mutant in which the D1 domain was replaced with a second copy of D2 (D2D2), a phosphatase-dead point mutant (C1142S; Streuli et al., 1990), and a mutation of the extracellular region known to disrupt binding to HSPGs (4K4A; Aricescu et al., 2002). A schematic view of these mutants is shown in Figure 5A.

Consistent with our hypothesis, we found that deletion of the PTPσ D2 domain completely abolished synaptogenic activity in the coculture assay with TrkC (Figures 5B,C). Surprisingly, however, we also found that loss of D1 abolished synaptogenic activity, but loss of phosphatase activity *via* the C1142S mutation had no effect. The D2D2 mutation also abolished synaptogenic activity, ruling out the possibility that the effects of the ΔD1 mutation were due to steric effects related to the position of the D2 domain relative to the cell membrane. Finally, we found no effect of the 4K4A mutation, indicating that the synaptogenic activity of PTPσ does not depend on extracellular HSPG binding. These effects were not due to differences in surface expression or local recruitment of the V5-PTPσ mutants since we stained for the extracellular V5 tag and chose fields for analysis based on equal surface levels (Supplementary Figure S2A). The lack of activity of the ΔD1 and D2D2 mutants is surprising given the high degree of 3D structural similarity of the wedge and D1 domains with the D2 domain. Aside from a small linker region between the wedge and D1 domain, the combined wedge and D1 structure overlaps almost perfectly with the D2 domain (Supplementary Figures S2B–D). Altogether, these data suggest that specific interactions with both the D1 and D2 domains of PTPσ are required for its function in presynaptic differentiation.

### Identification of PTPσ Point Mutants to Disrupt Specific Interactions

To further elucidate the mechanisms by which the PTPσ intracellular domain signals the formation of a new presynaptic compartment, we sought to identify mutations that would specifically disrupt the binding of PTPσ to one or another of its known intracellular interaction partners. We chose to use this approach rather than one based on knockdown of each individual interacting protein because it more directly addresses whether binding between PTPσ and the protein of interest is required, which is a different question from whether the interaction partner itself is required. While this work was in progress, it was shown that knockdown of liprin-α2 and liprin-α3 reduced the ability of both TrkC and NL2 to induce synapses in the coculture assay (Han et al., 2018). This result is consistent with the hypothesis that direct binding of PTPσ to liprin-α (Serra-Pagès et al., 1995), and indirect binding of neurexin to liprin-α through a mutual binding partner such as CASK (Hata et al., 1996; Olsen et al., 2005) might be a necessary step in the synaptogenic process. However, it is also consistent with alternative models in which liprin-α is either recruited to the synapse *via* other proteins with which it interacts or is required within the neuron but does not need to be recruited to nascent synapses.

We tested 22 candidate mutations consisting of between two and seven amino acids each which were well-conserved at least within mammalian LAR-RPTP family members and in many cases in *C. elegans* and *Drosophila* homologs as well. These candidate mutations were surface-accessible and located close to one another on the protein surface (Supplementary Figure S3). We also assayed the PPLL mutation which was previously shown to disrupt PTPσ homodimerization (Hofmeyer and Treisman, 2009). We screened these mutants for their ability to bind to liprin-α2, caskin1, trio, or to a second molecule of PTPσ. To do this, we used the DHFR protein complementation assay, in which the ability of yeast to grow in the presence of the drug methotrexate (MTX) is dependent on the amount of binding between two putative interacting proteins, each of which is fused to one of two fragments of an MTX-insensitive mutant of the DHFR enzyme (Tarassov et al., 2008; Rochette et al., 2015). We assessed the association of PTPσ or its intracellular domain fused to the DHFR N-terminal fragment with the interacting regions of liprin-α2, caskin1, and trio, as well as with PTPσ itself, fused to the DHFR C-terminal fragment. Negative control strains were constructed in which one of the two interacting proteins was replaced with either YFP or NgCAM. This maintained a constant size of the DHFR fusion proteins which, consistent with previous reports (Rochette et al., 2015), we found to be important for consistency of the assay. Growth of the resulting strains was measured in media containing either MTX (200 μg/mL, 1% DMSO) or DMSO alone. For each strain, an interaction score was calculated (see “Materials and Methods” section) such that a score of 0 indicates growth equivalent to that of the negative control strain, and 1 indicates WT growth.

Several mutations (MELEFK, WENNS, KLREMG, and HQYWP) moderately to severely disrupted all interactions tested and thus were considered nonspecific (interaction score <0.3 for at least three out of four ligands, shown in gray in Figure 6A). These mutations may disrupt activity in the DHFR assay by affecting protein folding, trafficking, or stability. Once these nonspecific mutations are ignored, mutations affecting binding to liprin-α2 and caskin1 each form a cluster on the surface of the protein (Figure 6A, top row). Binding of PTPσ to liprin-α2 is partially disrupted (with interaction scores between 0.21 and 0.37) by the closely spaced AEY, DWPE, QVHK, QFG, and MRYE mutations (blue). The caskin1 interaction is abolished completely by the MRYE and EGFID mutations (purple). Mutations on the opposite side of the protein, aside from those which were nonspecific, had minimal effect on binding of either protein to PTPσ (Figure 6A, bottom row). These results represent the gross mapping of binding sites on the PTPσ D2 domain for liprin-α2 and caskin1. The apparent liprin-α2 binding site on PTPσ constitutes a larger interaction surface than that for caskin1. It is noteworthy that these putative binding sites overlap, suggesting that simultaneous binding of PTPσ to liprin-α2 and caskin1 may not be possible. Supporting a competitive binding model, LAR is unable to act as a bridge between caskin and liprin-α in yeast two-hybrid assays (Weng et al., 2011). Based on the mutations we tested, clear binding sites were not seen in the D2 domain for PTPσ homodimerization or for trio. For homodimerization, this is not surprising given that binding is thought to be mediated by the wedge domain and the D1 domain (Hofmeyer and Treisman, 2009).

There were several mutations that primarily disrupted binding to liprin-α2, and of these QFG appeared to be the most specific, with an interaction score of 0.37, compared to between 0.77 and 0.88 for its interaction with caskin1, trio, and PTPσ WT (Figures 6B,C). The EGFID mutation is highly effective as well as specific in its disruption of caskin1 binding, with an interaction score of −0.11 for caskin1, and between 0.73 and 1.02 for the other interactions. These two mutations were chosen for further study to determine whether the binding sites on PTPσ for liprin-α, caskin, or both were required for its role in synaptogenesis. The PPLL mutation, which was previously reported to disrupt PTPσ homodimerization only when present on both copies of the protein (Hofmeyer and Treisman, 2009), did indeed disrupt activity in the DHFR assay. In contrast to the previous report, in the DHFR assay, we observed impaired binding between PTPσ WT and PPLL (interaction score of 0.46) as well as between PTPσ PPLL and PPLL (interaction score of 0.35). We also found that this mutation partially disrupts binding to caskin1 (interaction score of 0.51), although not as strongly as EGFID, and does not disrupt binding to liprin-α2.

### Co-clustering of PTPσ Mutants With Liprin-α2 in HEK Cells

Liprin-α has been previously shown to form clusters when transfected in cell lines and to recruit LAR-RPTPs to these clusters (Serra-Pagès et al., 1998). As a second measure of the interaction strength between the PTPσ mutants and liprin-α2, we co-transfected myc-liprin-α2 with V5-PTPσ WT or mutant as well as CFP as a cell fill into HEK 293 cells. In the absence of liprin-α2, PTPσ was mostly diffuse (Figure 7A, left-most column). Consistent with previous reports, liprin-α2 formed small puncta in some cells when transfected alone (Figure 7A, second column from left). When co-transfected with PTPσ, the distribution of liprin-α2 became more distinctly clustered. Notably, PTPσ was recruited to sites of liprin-α2 clustering (Figure 7A, third column from left). To quantitate these interactions, we calculated a ratio between the intensity of PTPσ fluorescence within the liprin-α2 patches and that in the remainder of the cell. Analogous experiments were not possible for caskin1, trio, or PTPσ homodimerization because they did not exhibit the same co-clustering phenomenon.

When we transfected HEK cells with liprin-α2 together with mutant versions of PTPσ, we found that the ΔICR, ΔD2, and QFG mutants disrupted the co-clustering of the two proteins, such that the distribution of both resembled that seen in singly-transfected cells. The PPLL and EGFID mutants were essentially unaffected, and the ΔD1 and D2D2 mutants showed a partial disruption of the co-clustering phenomenon. Visually, the liprin-α2 clusters in cells co-transfected with ΔD1 or D2D2 appeared intermediate between the WT and ΔICR forms, and the mutant PTPσ was recruited to these patches, though to a lesser extent than with the WT protein. Quantification also revealed an intermediate phenotype. When recruitment was measured based on total PTPσ staining both ΔD1 and D2D2 were statistically different from WT but not ΔICR, whereas when surface PTPσ was measured, the ΔD1 mutant was significantly different from ΔICR but not WT (Figures 7B,C). These results are consistent with a model in which binding between liprin-α and PTPσ is mainly mediated by the PTPσ D2 domain as previously reported (Serra-Pagès et al., 1995) and involve the QFG residues, but also involves some contribution from the D1 domain. Thus, D1 and D2 together mediate full-strength binding of PTPσ to liprin-α.

### Recruitment of Liprin-α2 to Nascent Synapses in the Presence of PTPσ Mutants

**Figure 8:**
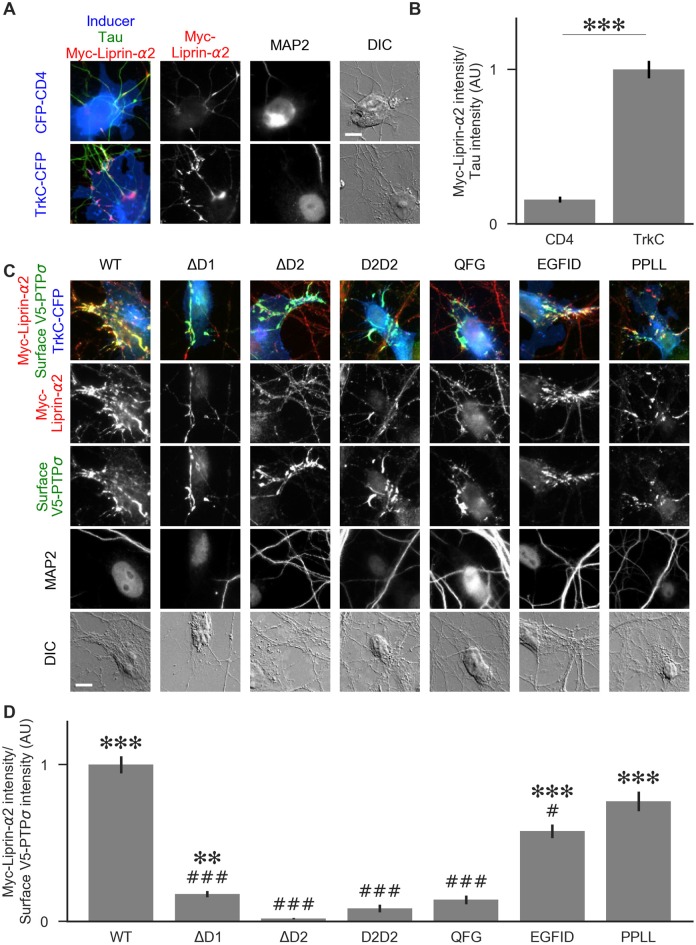
Recruitment of liprin-α2 by TrkC coculture in the presence of PTPσ mutants. **(A)** Representative images of cocultures with either CFP-CD4 or TrkC-CFP in which neurons were transfected with and stained for myc-liprin-α2. Transfections were performed at DIV 0 and coculture assays were performed at DIV 13–14. The transfected protein was recruited by COS cells expressing TrkC but not by those expressing CD4. MAP2 labeling of dendrites was used to exclude native synapses from analysis. Scale bar represents 10 μm. **(B)** Quantification of recruitment shown in **(A)**. Measurement was performed on the contact area only, defined as the region where axons contact the inducer-expressing COS cell, excluding the area overlapped by MAP2-positive dendrites. Values are normalized to the mean value of recruitment by TrkC from the same culture. Data are mean ± SEM. ***p < 0.001, Mann-Whitney, *n* = 96 cells per condition from three independent cultures. **(C)** Representative images of neurons co-transfected with myc-liprin-α2 and V5-PTPσ WT or mutant, treated with shPTP-expressing AAVs at DIV 6, and cocultured with COS cells expressing TrkC. Scale bar represents 10 μm. **(D)** Quantification of the extent of myc-liprin-α2 recruitment relative to V5-PTPσ recruitment as shown in **(C)**. There were no differences in surface levels of the V5-PTPσ mutants in the areas assayed (Supplementary Figure S2E). Measurements were limited to the area of the COS cell which lacked MAP2 signal. All values are normalized to the mean of the WT condition from the same culture. Data are expressed as mean ± SEM. Overall *p*-value for this experiment was 3.43 × 10^–39^, Kruskal-Wallis, *n* = 20–50 cells per condition from two cultures. ***p < 0.001, ***p < 0.01 compared to ΔD2; ^###^*p* < 0.001, ^#^*p* < 0.05 compared to WT, Dunn’s *post hoc* test.

We predicted that the same PTPσ mutants which disrupted binding to liprin-α in yeast or in HEK cells would also impair recruitment of liprin-α to sites of presynaptic differentiation. To visualize recruitment of liprin-α to presynaptic sites, we transfected neurons with myc-liprin-α2, along with V5-PTPσ, at the time of plating. When we performed cocultures with neurons treated in this manner, we observed significant recruitment of myc-liprin-α2 to COS cells expressing TrkC, compared to those expressing the control protein CD4 (Figures 8A,B).

To visualize liprin-α recruitment by PTPσ mutants, we performed additional cocultures in the same manner, except that neurons were treated with shPTP to remove native LAR-RPTPs. In addition to WT PTPσ, we tested the ΔD1, ΔD2, D2D2, QFG, EGFID, and PPLL mutants. As in Figure 5, we selected cells such that the surface levels of V5-PTPσ were equal across conditions (Supplementary Figure S2E). We found that ΔD1, ΔD2, D2D2, and QFG all reduced the extent of liprin-α2 recruitment substantially. Although ΔD1 mediated very weak recruitment, it was significantly higher than that of ΔD2 (Figures 8C,D). The level of recruitment by PPLL was similar to WT. Recruitment by EGFID was significantly different from both WT and ΔD2, although it was much closer to WT. These results suggest that direct binding between PTPσ and liprin-α is required for liprin-α to be recruited to a nascent synapse and that indirect interactions through mutual binding partners are not sufficient.

### Synaptogenic Activity of PTPσ Point Mutants

**Figure 9:**
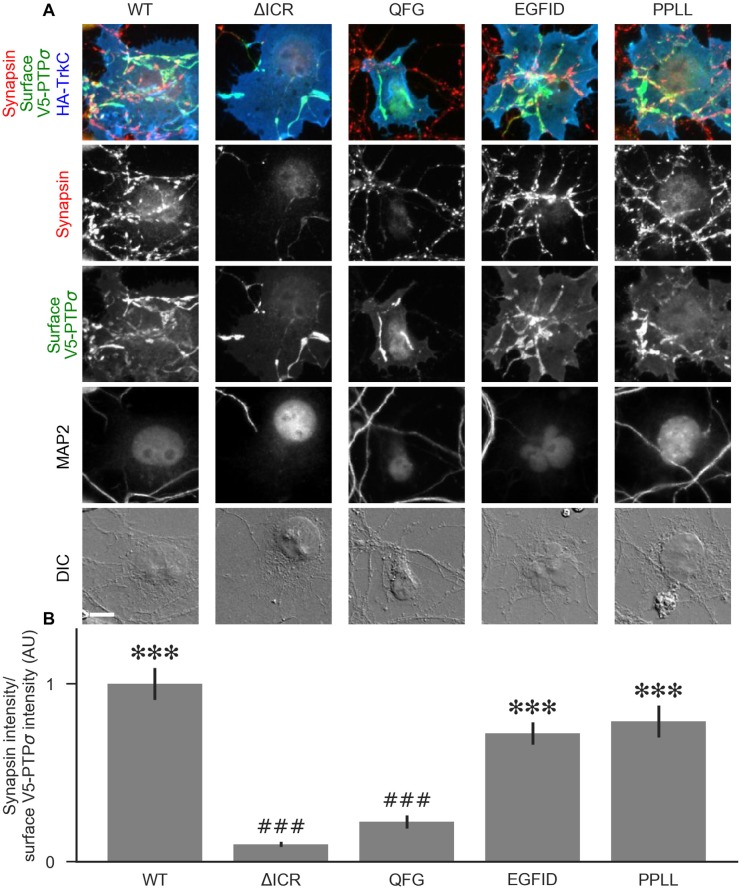
The liprin-α2 binding site, but not the caskin1 binding site or homodimerization site, of PTPσ is required for TrkC synaptogenic activity. **(A)** Representative images of HA-TrkC cocultures where neurons were treated with shPTP-expressing AAVs, and rescued using RNAi-resistant V5-PTPσ carrying the indicated mutations. Rescue constructs were introduced by nucleofection at DIV 0, AAV shRNAs were applied at DIV 6, and coculture assays were performed at DIV 13–14. The V5-PTPσ constructs were recruited by TrkC-expressing COS cells, in some cases inducing local clustering of synapsin. MAP2 labeling of dendrites was used to exclude native synapses from analysis. The QFG, EGFID, and PPLL mutations were shown to primarily disrupt binding of PTPσ to liprin-α2, caskin1 and to itself, respectively (see Figures 6, 7). Scale bar represents 10 μm. **(B)** Quantification of induced synapsin clustering in **(A)**. There were no differences in surface levels of the V5-PTPσ mutants (Supplementary Figure S2F). Measurements were limited to the area of the COS cell which lacked MAP2 signal. All values are normalized to the mean of the WT condition from the same culture. Data are expressed as mean ± SEM. Overall *p*-value for this experiment was 9.86 × 10^−31^, Kruskal-Wallis, *n* = 34–71 cells per condition from three cultures. ***p < 0.001 compared to ΔICR, ^###^*p* < 0.001 compared to WT, Dunn’s *post hoc* test.

To determine the relative contributions to PTPσ’s synaptogenic activity of the binding interactions between PTPσ and liprin-α, caskin, and itself, we used the QFG, EGFID, and PPLL mutants to rescue activity in the coculture assay. Transfection of shRNA-resistant V5-PTPσ, infection by shPTP, and coculture with HA-TrkC were performed as in Figure 5. Fields were selected in order to ensure equal levels of axonal surface V5-PTPσ in contact with the TrkC-expressing COS cells (Supplementary Figure S2F), and recruitment of synapsin was measured. We found that the QFG mutation reduced synapsin clustering to levels similar to the ΔICR mutant (Figure 9). In contrast, synapsin clustering by the PPLL and EGFID mutants was not significantly different from WT. Taken together, our results revealed a parallel in the extent of interaction with liprin-α2 in the yeast and HEK cell assays (Figures 6, 7), recruitment of myc-liprin-α2 in neurons (Figure 8), and presynaptic differentiation (Figures 5, 9). Thus, binding of PTPσ to liprin-α is likely essential for synaptogenesis induced by the TrkC/PTPσ trans-synaptic complex. In contrast, mutations which disrupt PTPσ homodimerization and binding to caskin had minimal effect on PTPσ-mediated presynaptic differentiation.

## Discussion

The results presented here provide insight into the roles of the LAR-RPTP family in presynaptic differentiation. The ability of the TrkC/PTPσ complex to induce new presynaptic sites was not impaired by mutations which disrupted PTPσ homodimerization, phosphatase activity, or binding to HSPGs or to caskin. In contrast, we found that PTPσ-mediated presynaptic differentiation requires liprin-α-binding regions in the PTPσ D2 and D1 domains. While this article was in preparation, Han et al. (2018) reported an overlapping study, but with some important differences. Their findings are consistent with ours regarding the importance of liprin-α but diverge regarding the role of PTPσ’s phosphatase activity.

### The LAR-RPTP Family Functions in Axon Growth

We observed a reduction in axon outgrowth by neurons treated with shPTP compared to those treated with shCtrl, with no difference in dendritic outgrowth. The inability of V5-PTPσ to rescue this phenotype could result both from technical factors, such as the electroporation method used, which only targets a portion (~50%) of the cells, or from off-target effects of the shRNA on neuronal process growth. Alternatively, the lack of rescue could result from the fact that we used only a single LAR-RPTP family member. In other words, the reduction in growth might be caused primarily by the loss of LAR and/or PTPδ, rather than by loss of PTPσ. Whereas TrkC-mediated presynaptic differentiation assessed in the other experiments involves only PTPσ (Takahashi et al., 2011), much evidence indicates a role of all three LAR-RPTPs in axon outgrowth, targeting, and regeneration (Chagnon et al., 2004; Stoker, 2015). Furthermore, the roles of LAR-RPTPs in regulating axon growth may be context dependent. PTPσ knockout mice have shown accelerated axon outgrowth following nerve injury and in cultures of cortical or dorsal root ganglion neurons (McLean et al., 2002; Thompson et al., 2003; Sapieha et al., 2005; Siu et al., 2007) and increased hippocampal mossy fiber sprouting with aging or seizures (Horn et al., 2012). In contrast, disrupting the function of CRYPα, the chicken homolog of PTPσ, inhibited retinal axon outgrowth (Ledig et al., 1999). Uninjured PTPσ knockout mice show a thinner corpus callosum compared to WT controls, which could indicate defects in either outgrowth or targeting (Meathrel et al., 2002). The extracellular region of PTPσ can bind to both HSPGs and chondroitin sulfate proteoglycans (CSPGs), and its status as an activator or inhibitor of axon outgrowth appears to depend on the local balance of HSPGs and CSPGs (Coles et al., 2011). Interestingly, the extracellular ligand needed for CRYPα-dependent promotion of axon outgrowth, although it was not identified specifically, was determined to come from glial endfeet (Ledig et al., 1999). It is possible that the culture system used in our experiments, in which neurons contact substrates coated with glial-derived factors, results in an environment in which the ligands that PTPσ (and possibly PTPδ and LAR) are exposed to are, on the whole, growth-promoting rather than growth-inhibiting.

Whatever the reason for the reduction in axon outgrowth with shPTP, this change in neuronal morphology would complicate the interpretation of many assays for native synapses. Any potential reductions in synapse density could result from either a reduction in axon/dendrite contacts as a result of reduced axon length overall, or from specific roles of the LAR-RPTPs in local differentiation of contacts into functional synapses. Thus, the reductions in synapse density and in the frequency of miniature synaptic currents reported with knock-down of LAR-RPTPs in hippocampal cultures (Han et al., 2018) could reflect deficits in axon outgrowth and/or in synapse development.

Effects of the shPTP treatment on axon outgrowth were also apparent in the diminished level of axon recruitment in the coculture experiments. Expression of shRNA-resistant V5-PTPσ partially rescued axon recruitment, to a greater extent for cells expressing TrkC than NGL-3 or NL2. In the statistical analyses, axon recruitment was significantly diminished in the shPTP knockdown and PTPσ rescue group compared with the shCtrl group only for NL2 and not for TrkC or NGL-3. Considering that NL2 is not a LAR-RPTP ligand, these effects on axon recruitment in coculture are likely due to the effects of shPTP treatment on axon growth in general. Thus to specifically assay synaptogenic activity, we controlled for differences in axon recruitment in the coculture assays.

### Role of LAR-RPTPs in Synaptogenesis

We found that LAR-RPTP knockdown abolished the synaptogenic coculture activity of both TrkC and NGL-3 almost entirely, whereas there was no effect on the activity of the neurexin ligand NL2. Our findings contrast with those of another recent study, which found no effect on coculture with NGL-3 in response to knockdown of any or all of the LAR-RPTPs (Han et al., 2018). It could be that NGL-3’s activity requires a fairly low threshold amount of LAR-RPTPs and that the extent of knockdown differed between the two studies. The finding that loss of the LAR-RPTPs is sufficient to abolish NGL-3 activity is consistent with the observation that NGL-3 binds to all three LAR-RPTP family members (Kwon et al., 2010) and has no other known extracellular binding partners. In the case of TrkC, we observed an increase in clustering of synapsin in response to transfection with V5-PTPσ relative to control V5-CD4, although this difference was not significant. This could indicate that the total level of LAR-RPTPs in untreated neurons is not saturating with respect to presynaptic differentiation induced by TrkC.

Despite compelling evidence for a central role of LAR-RPTPs in synaptogenesis in invertebrate systems (Kaufmann et al., 2002; Ackley et al., 2005), assessing their roles in native synaptogenesis in mammals has been more difficult, confounded in part by their roles in axon growth. Mice lacking PTPσ show increased hippocampal synapse density which may be related to increased axon growth (Horn et al., 2012). Importantly, these PTPσ deficient mice also show differences in synapse properties, including elevated paired-pulse facilitation suggesting a reduced probability of release, and reduced long term potentiation (Horn et al., 2012). TrkC-PTPσ and NGL-3-LAR-RPTP complexes may contribute to these synapse properties, and potentially to synapse density as indicated by deficits upon knockdown of TrkC or NGL-3 (Woo et al., 2009; Takahashi et al., 2011).

### Domain Requirements for Liprin-α Binding to PTPσ

Since no binding partners of the D1 domain besides PTPσ itself are known, our initial hypothesis was that the D1 domain would either be dispensable for synaptogenic activity or would be required for its phosphatase activity or for homodimerization. The finding that the ΔD1 mutation abolished coculture activity, but the C1142S phosphatase-dead and PPLL non-homodimerizing mutations had no effect, was surprising. Another hypothesis which we considered was that the D1 domain was not required for any catalytic or binding function, but instead acted as a spacer which positioned the D2 domain some distance from the plasma membrane, allowing for the formation of multi-protein complexes required for synapse formation. This hypothesis also turned out to be incorrect, as the D2D2 mutation in which the D1 domain was replaced by a second copy of the similarly-sized D2 domain also abolished activity. Thus, it appeared that the D1 domain must be performing some function which was previously unknown. One clue to this puzzle came from the fact that the ΔD1 mutant surprisingly showed a phenotype intermediate between WT and ΔICR when we tested its ability to co-cluster with liprin-α2 in HEK cells. Similarly, replacement of the D1 domain by a second D2 domain (D2D2) disrupted the ability of PTPσ to co-cluster with liprin-α2 in HEK cells. Furthermore, the ΔD1 and D2D2 mutants were deficient at recruiting liprin-α2 to TrkC-induced presynaptic sites in neurons. Our data are consistent with a model in which binding to liprin-α2 is primarily mediated by the D2 domain of PTPσ, but the D1 domain is required for full-strength binding. Binding between LAR and liprin-α was previously reported to be mediated by the D2 domain, based on the observation that the isolated D2 domain but not the isolated D1 domain showed interaction with liprin-α in a yeast two-hybrid assay (Serra-Pagès et al., 1995). However, this does not rule out the possibility that D1 contributes to the strength of the interaction.

### PTPσ-Liprin-α Interaction in Presynaptic Differentiation

A major aim of this study was to examine the downstream pathways and mechanisms which might mediate the synaptogenic effects of PTPσ’s interactions with its postsynaptic ligands. To test the functional relevance of PTPσ’s ability to bind liprin-α, caskin, and itself, we employed a series of point mutants identified using a protein complementation assay. Unfortunately, we were not able to test the importance of a third D2-interacting protein, trio, using this strategy because we did not find any appropriate point mutants. This experiment pointed to liprin-α as the likely most important downstream interacting partner of PTPσ. The QFG mutant, which was able to bind caskin and to homodimerize with PTPσ WT, but not bind liprin-α, almost completely abolished coculture activity. The EGFID and PPLL mutants, which disrupted caskin binding and homodimerization respectively, had mild if any effects.

Our results agree with those of Han et al. (2018) in identifying liprin-α as a likely mediator of PTPσ’s synaptogenic effects. If binding between PTPσ and liprin-α is required, one would expect that both proteins themselves would be necessary for PTPσ-mediated synaptogenesis. Han et al. (2018) found that knockdown of either PTPσ or of liprin-α2 and -α3 impaired the ability of PTPσ ligands to induce synaptogenesis in the coculture assay. Our work corroborates and extends upon this finding by providing evidence that not only are LAR-RPTPs and liprin-α required for synaptogenesis but that these two proteins must be able to bind one another.

We did not find any effect of the extracellular 4K4A mutation on coculture activity, indicating that PTPσ’s ability to bind HSPGs is not required for its synaptogenic effects, at least when triggered by binding to TrkC. Homodimerization *via* the ICR of PTPσ also appeared to be dispensable, based on the ability of the non-homodimerizing PPLL mutant to induce clustering of both liprin-α2 and synapsin. However, this does not preclude the possibility that indirect multimerization of PTPσ might be necessary. The PTPδ postsynaptic ligand SALM5 has been shown to induce formation of tetramers containing two molecules each of PTPδ and SALM5, independently of any direct interactions between PTPδ molecules (Goto-Ito et al., 2018; Lin et al., 2018).

We also found no effect of the phosphatase-dead C1142S mutant on coculture activity of TrkC, indicating that dephosphorylation of targets is not necessary for PTPσ to mediate presynaptic differentiation. In contrast to this finding, Han et al. (2018) reported that the same mutation (C1157S in their PTPσ construct) impairs or abolishes coculture activity of Slitrk1 and TrkC. Two potentially important methodological differences between our study and Han et al. (2018) may contribute to these differences in findings. First, we assessed coculture regions with equal surface expression of the various PTPσ constructs, as differences in surface trafficking could confound measures of presynaptic differentiation. Second, we excluded native synapses (synapsin puncta associated with MAP2-positive dendrites) from our measures and quantified only presynaptic sites induced by contact with TrkC-expressing cells. This issue may be particularly significant given the finding that PTPσ phosphatase activity regulates native synapse density in hippocampal cultures through a postsynaptic mechanism (Dunah et al., 2005).

In summary, our results indicate that liprin-α binding is likely required for PTPσ-mediated presynaptic differentiation, and that caskin binding, homodimerization, phosphatase activity, and HSPG binding are all dispensable. We cannot be certain that the PTPσ mutations used in our molecular replacement experiments did not disrupt multiple interactions, including potentially those with as yet unidentified binding partners of PTPσ. However, the correspondence between those mutations which disrupted binding to liprin-α in yeast and/or HEK cells and those which abolished recruitment of both liprin-α2 itself and synapsin in the coculture assay is consistent with the hypothesis that binding to liprin-α is necessary for PTPσ to induce presynaptic differentiation.

## Data Availability

The raw data supporting the conclusions of this manuscript will be made available by the authors, without undue reservation, to any qualified researcher. The datasets generated for this study are available on request to the corresponding author.

## Ethics Statement

This study was carried out in accordance with the recommendations of the Canadian Council on Animal Care. The protocol was approved by the University of British Columbia Animal Care Committee.

## Author Contributions

CB and AC conceived the project and wrote the article with assistance from all authors. NP, BK, and TS performed confirmation of viral-mediated knockdowns and YG did some preliminary characterization. CB generated and analyzed all other data. CL and JC provided expertise and training for the yeast DHFR assay.

## Conflict of Interest Statement

The authors declare that the research was conducted in the absence of any commercial or financial relationships that could be construed as a potential conflict of interest.
