# Supplementary material for: Mechanisms of PTPσ-Mediated Presynaptic Differentiation
Source: Front Synaptic Neurosci. 2019 May 22;11:17. doi: 10.3389/fnsyn.2019.00017 (PMC6540616; doi:10.3389/fnsyn.2019.00017)
Supplement: Supplementary file 1 [file Data_Sheet_1.PDF]

Supplementary Material

1.1 Supplementary Figures

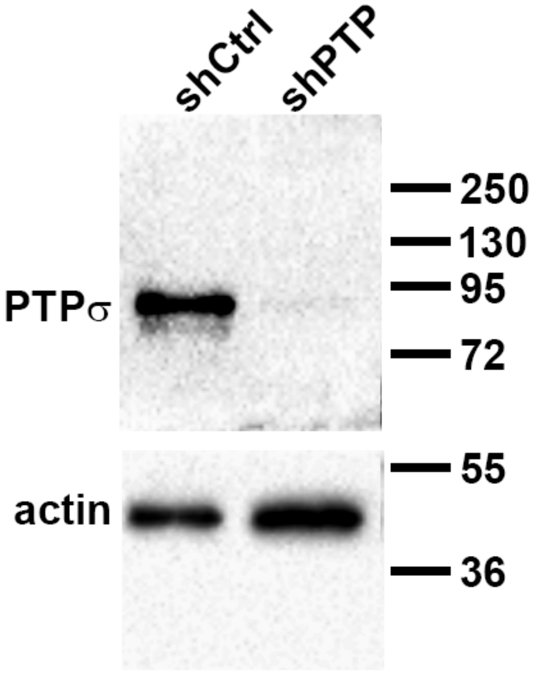

Supplementary Figure 1. Full-size blot comparing LAR-RPTP levels in neurons treated with shCtrl versus shPTP (related to Figure 1)

The membrane was cut into two pieces, and the upper and lower portions were probed for PTPσ and β-actin, respectively.

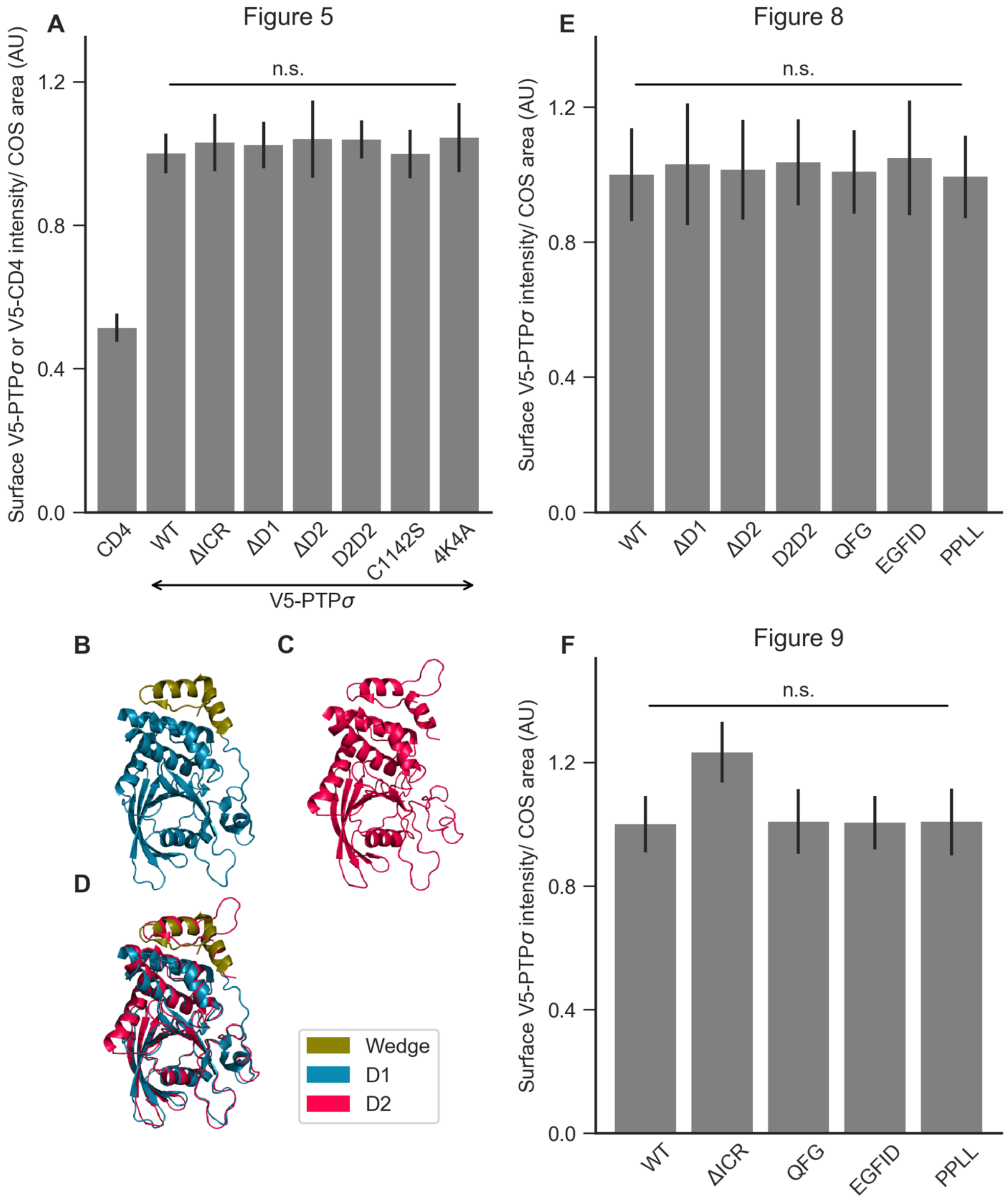

Supplementary Figure 2. Quantification of V5-PTPσ surface intensity, and structural alignment of D1 and D2 domains (related to Figures 5, 8, and 9).

(**A**) Quantification of surface V5-PTPσ WT or mutant recruited to TrkC-expressing COS cells. Values are normalized to the mean of the wild-type (WT) condition from the same culture. Overall p-value (excluding CD4) was 0.44, Kruskal-Wallis. (**B-D**) Alignment of the wedge + D1 domains with

the D2 domain of PTPσ (3SR9; Hou et al. 2011) showing their 3-dimensional similarity. Isolated wedge + D1 and D2 domains are shown in D and E, respectively, in the same orientation as in the aligned image in F. Note the gap in panel C (right side of molecule), indicating that the D1 wedge domain is positioned differently from its counterpart in the D2 domain. (**E**) Quantification of surface V5-PTPσ WT or mutant recruited to COS cells expressing TrkC in the liprin-α2 recruitment experiment shown in Figure 8. Values are normalized to the mean of the WT condition from the same culture. Overall p-value was 0.56, Kruskal-Wallis. (**F**) Quantification of surface V5-PTPσ WT or mutant recruited to COS cells expressing TrkC in the presynaptic induction experiment shown in Figure 9. Values are normalized to the mean of the WT condition from the same culture. Overall p-value was 0.17, Kruskal-Wallis.

```
Fly PTP69D     1 EEEDSVSIYNTVCDLRHQRNFLVQSLKQYIFLYRALLDTGTFGNTDICIDTMASAIESLKR-KPNEGKCK
Worm_PTP-3     1 RYDNTVDIYGCVTALRAQRSYMVQTEEQYIFIHDAVLDAVNSGSTEVPASRLHQHLHILSQ-PSADQLSG
Fly LAR        1 KHEKIIDIYGHVTCLRAQRNYMVQTEDQYIFIHDAILEAICGVTEVPARNLHTHLQKLLITEPGETISG
Human_LAR      1 KHEKTVDIYGHVTCMRSQRNYMVQTEDQYVFIHEALLEAATCGHTEVPARNLYAHIQKLGQVPPGESVTA
Mouse LAR      1 KHEKTVDIYGHVTCMRSQRNYMVQTEDQYVFIHEALLEAAMCGHTEVLARNLYAHIQKLGQVPPGESVTA
Human_PTPRD    1 KHEKTVDIYGHVTLMRAQRNYMVQTEDQYIFIHDALLEAVTCGNTEVPARNLYAYIQKLTQIETGENVTG
Mouse PTPRD    1 KHEKTVDIYGHVTLMRAQRNYMVQTEDQYIFIHDALLEAVTCGNTEVPARNLYAYIQKLTQIETGENVTG
Human_PTPRS    1 KPEKTVDVYGHVTLMRSQRNYMVQTEDQYSFIHEALLEAVGCGNTEVPARSLYAYIQKLAQVEPGEHVTG
Mouse PTPRS    1 KTEKTVDVYGHVTLMRSQRNYMVQTEDQYGFIHEALLEAVGCGNTEVPARSLYTYIQKLAQVEPGEHVTG
Name                                     TED                    PARS YTYIQ      PGE
Mutate to                                AAA                    AAAA ATYIA      AAA
Name                                                                   YIQK
Mutate to                                                              AIQA

Fly PTP69D    70 LEVEFEKLLATADEISKSCSVGENEENNMKNRSQEIIPYDRNRVILTPLPMRENSTYINASFIEGYDNSE
Worm_PTP-3    70 IDMEF-RHLTTLKWTSNRCTVANLPVNRPKNRMLSAVPYDSNRVIMRLLPGADGSDYINASWIDGYKERG
Fly LAR       71 MEVEF-KKLSNVKMDSSKFVTANLPCNKHKNRLVHILPYESSRVYLTPIHGIEGSDYVNASFIDGYRYRS
Human_LAR     71 MELEF-KLLASSKAHTSRFISANLPCNKFKNRLVNIMPYELTRVCLQPIRGVEGSDYINASFLDGYRQQK
Mouse LAR     71 MELEF-KLLANSKAHTSRFVSANLPCNKFKNRLVNIMPYELTRVCLQPIRGVEGSDYINASFLDGYRQQK
Human_PTPRD   71 MELEF-KRLASSKAHTSRFISANLPCNKFKNRLVNIMPYESTRVCLQPIRGVEGSDYINASFIDGYRQQK
Mouse PTPRD   71 MELEF-KRLASSKAHTSRFISANLPCNKFKNRLVNIMPYESTRVCLQPIRGVEGSDYINASFLDGYRQQK
Human_PTPRS   71 MELEF-KRLANSKAHTSRFISANLPCNKFKNRLVNIMPYESTRVCLQPIRGVEGSDYINASFIDGYRQQK
Mouse_PTPRS   71 MELEF-KRLASSKAHTSRFITASLPCNKFKNRLVNILPYESSRVCLQPIRGVEGSDYINASFIDGYRQQK
Name             MELEF-K                 PCNK                    IRGVE
Mutate to        AAAAA-A                 AANA                    AAAAA

Fly PTP69D   140 TFIIAQDPLENTIGDFWRMISEQSVTTLVMISEIGD-GPRKCPRYW-ADDEVQYDHILVKYVHSESCPYY
Worm_PTP-3   139 AYIATQAPTNETAADFWRAIWEHNSPIIAMLVRTNERGQEQCSDYWPLETGVQVGMLVVEPMAEYDMKHY
Fly LAR      140 AYIAAQGPVQDAAEDFWRMLWEHNSTIVVMLTKLKEMGREKCFQYWPHERSVRYQYYVVDPIAEYNMPQY
Human_LAR    140 AYIATQGPLAESTEDFWRMLWEHNSTIIVMLTKLREMGREKCHQYWPAERSARYQYFVVDPMAEYNMPQY
Mouse_LAR    140 AYIATQGPLAESTEDFWRMLWEHNSTIIVMLTKLREMGREKCHQYWPAERSARYQYFVVDPMAEYNMPQY
Human_PTPRD  140 AYIATQGPLAETTEDFWRMLWEHNSTIVVMLTKLREMGREKCHQYWPAERSARYQYFVVDPMAEYNMPQY
Mouse PTPRD  140 AYIATQGPLAETTEDFWRMLWEHNSTIVVMLTKLREMGREKCHQYWPAERSARYQYFVVDPMAEYNMPQY
Human_PTPRS  140 AYIATQGPLAETTEDFWRMLWENNSTIVVMLTKLREMGREKCHQYWPAERSARYQYFVVDPMAEYNMPQY
Mouse_PTPRS  140 AYIATQGPLAETTEDFWRALWENNSTIVVMLTKLREMGREKCHQYWPAERSARYQYFVVDPMAEYNMPQY
Name                                 WENNS       KLREMG    HQYWP          VVDP AEY
Mutate to                            AAAAA       AAAAAA    AAYAA          AVAA GAA

Fly PTP69D   208 TRREFYVTNCKIDDTLKVTQFQYNGWPTVDGEVPEVCRGIIELVDQAYNHYKNNKNSGCRSPITVHCSLG
Worm_PTP-3   209 HLREFRISDINTREVRTVRQFHFMEWP--DVGKPHTADHFLDFVTQVHNTYAQF---GCTGPITVHCCSG
Fly LAR      210 KLREFKVTDARDGSSRTVRQFQFIDWP--EQGVPKSGEGFIDFIGQVHKTKEQF---GQDGPITVHCSAG
Human LAR    210 ILREFKVTDARDGQSRTIRQFQFTDWP--EQGVPKTGEGFIDFIGQVHKTKEQF---GQDGPITVHCSAG
Mouse_LAR    210 ILREFKVTDARDGQSRTIRQFQFTDWP--EQGVPKTGEGFIDFIGQVHKTKEQF---GQDGPITVHCSAG
Human_PTPRD  210 ILREFKVTDARDGQSRTVRQFQFTDWP--EQGVPKSGEGFIDFIGQVHKTKEQF---GQDGPISVHCSAG
Mouse_PTPRD  210 ILREFKVTDARDGQSRTVRQFQFTDWP--EQGVPKSGEGFIDFIGQVHKTKEQF---GQDGPISVHCSAG
Human PTPRS  210 ILREFKVTDARDGQSRTVRQFQFTDWP--EQGVPKSGEGFIDFIGQVHKTKEQF---GQDGPISVHCSAG
Mouse_PTPRS  210 ILREFKVTDARDGQSRTVRQFQFTDWP--EQGAPKSGEGFIDFIGQVHKTKEQF---GQDGPISVHCSAG
Name                                     DWP--E       EGFID   QVHK   QF---G
Mutate to                                AWA--A       AGFAA   AVAA   AA---A

Fly PTP69D   278 TDRSSIFVAMCILVQHLRLEKCVDICATTRKLRSQRTGLINSYAQYEFLHRAIINY-SDLHHIAESTLD
Worm_PTP-3   274 AGRTAVFIALSIILDRMRAEHVVDVFTTVKLLRTERQNMIQEPEQYHFLYLAAYEYLAAYDNFS-----
Fly LAR      275 VGRSGVFITLSIVLERMQYEGVLDVFQTVRILRSQRPAMVQTEDQYHFCYRAALEYLGSFDNYTN----
Human LAR    275 VGRTGVFITLSIVLERMRYEGVVDMFQTVKTLRTQRPAMVQTEDQYQLCYRAALEYLGSFDHYAT----
Mouse LAR    275 VGRTGVFITLSIVLERMRYEGVVDMFQTVKTLRTQRPAMVQTEDQYQLCYRAALEYLGSFDHYAT----
Human_PTPRD  275 VGRTGVFITLSIVLERMRYEGVVDIFQTVKMLRTQRPAMVQTEDQYQFSYRAALEYLGSFDHYAT----
Mouse PTPRD  275 VGRTGVFITLSIVLERMRYEGVVDIFQTVKMLRTQRPAMVQTEDQYQFCYRAALEYLGSFDHYAT----
Human PTPRS  275 VGRTGVFITLSIVLERMRYEGVVDIFQTVKMLRTQRPAMVQTEDEYQFCYQAALEYLGSFDHYAT----
Mouse_PTPRS  275 VGRTGVFITLSIVLERMRYEGVVDIFQTVKVLRTQRPAMVQTEDEYQFCFQAALEYLGSFDHYAT----
Name                             MRYE            RTQRPAMVQ          AALEYLG
Mutate to                        AAAA            ATQRAGMVA          GGAAAAA
```

Supplementary Figure 3. LAR-RPTP alignment showing point mutants tested in the DHFR assay (related to Figure 6).

Alignment of LAR-RPTP C-terminal sequences from human, mouse, worm, and fly including part of the D1 domain, the entire D2 domain, and the C-terminus. Mutations used in the DHFR assay (except for PPLL, which is N-terminal to the sequence shown here) are indicated below the alignment. Dashes indicate a single mutation (eg MELEFK) and spaces indicate two separate mutations (eg PARS and YTYIQ). Mutations shown in bold are those used in the coculture assay. Alignment was created using BOXSHADE.
